# The Subliminal Threshold Estimation Procedure (STEP): A calibration method tailored for estimating subliminal thresholds

**DOI:** 10.3758/s13428-025-02872-3

**Published:** 2025-12-01

**Authors:** Eden Elbaz, Itay Yaron, Liad Mudrik

**Affiliations:** 1 Sagol School of Neuroscience, Tel-Aviv University, Tel-Aviv, Israel; 2 School of Psychological Sciences, Tel-Aviv University, Tel-Aviv, Israel; 3 Brain, Mind, and Consciousness Program, Canadian Institute for Advanced Research, Toronto, ON, Canada

**Keywords:** Unconscious processing, Calibration, Subliminal threshold, Psychophysics, Threshold estimation, Masking

## Abstract

A major challenge in studying unconscious processing is to effectively suppress the critical stimulus while allowing maximal signal strength for adequate sensitivity to detect an effect, if it exists. A possible way to do this is to calibrate stimulus strength. While calibrating stimulus strength is common in psychophysics, current calibration methods are not designed to find the maximal intensity in which the stimulus can still be rendered unconscious (i.e., find the upper subliminal threshold for each participant). Here, we demonstrate how calibration can be utilized to estimate, for each observer, this targeted threshold. We present a novel calibration procedure: the Subliminal Threshold Estimation Procedure (STEP), specifically designed for estimating the upper subliminal threshold for each individual. Using simulations, we showed that STEP outperforms existing calibration methods, which yielded strikingly low accuracy. We then further validated STEP using three empirical experiments. Together, these results establish STEP as highly beneficial for the study of unconscious processing.

**Supplementary Information:**

The online version contains supplementary material available at 10.3758/s13428-025-02872-3.

## Introduction

The scope of unconscious processing has been investigated in psychological research for decades. Numerous studies have attempted to examine whether unconscious processing exists, and if so, what its limits are, often yielding conflicting results (Hassin, 2013; Hesselmann & Moors, 2015; Kouider & Dehaene, 2007; Mudrik & Deouell, 2022). While some studies provided evidence supporting unconscious processing across various cognitive domains (e.g., Dehaene et al., 1998; Kouider & Dupoux, 2005; Lau & Passingham, 2007; Van Opstal et al., 2011; Vorberg et al., 2003), others failed to find evidence for such high-level processing without awareness or questioned the validity of existing findings, either failing to replicate them (Andrews et al., 2011; Biderman & Mudrik, 2018; Chien et al., 2023; Heyman & Moors, 2014; Stein et al., 2020) or raising different methodological limitations (Meyen et al., 2022; Shanks, 2017; Vermeiren & Cleeremans, 2012; Zerweck et al., 2021).

Different explanations can account for these conflicting findings. On the one hand, positive findings might stem from methodological problems (Holender, 1986; Meyen et al., 2022; Pratte & Rouder, 2009; Vermeiren & Cleeremans, 2012; Zerweck et al., 2021). One prominent problem refers to the common practice of excluding trials in which participants might have seen the stimulus to some extent (Biderman & Mudrik, 2018; Hesselmann et al., 2016; Huang et al., 2014; Kiefer, 2019). This is done using trial-by-trial subjective measures, where participants provide direct reports of their experience. Such post hoc trial exclusion is motivated by the assumption that the remaining trials provide a sample that is uncontaminated by conscious processing. Similarly, researchers often exclude participants who show above chance objective performance; There, participants’ awareness of the stimulus is evaluated based on their performance in a direct detection/discrimination forced-choice task about the stimulus (e.g., where it appeared, what its category was, etc.). The underlying assumption is that such performance relies on consciously processed information (for criticism, see Ko & Lau, 2012; Reingold & Merikle, 1988). Thus, any significant deviation from chance performance is taken as evidence for some conscious perception of the stimulus.

Critically however, both types of post-hoc data exclusion evoke regression to the mean (RTTM), consequently leading to underestimation of awareness, and potentially inflating the observed unconscious processing effect due to conscious processing contamination (Dienes, 2024; Fahrenfort et al., 2024; Shanks, 2017). Trial exclusion might also affect the reliability and sensitivity of the objective measure, as it might lead to having too few trials to gain sufficient power for detecting above-chance performance in the objective measure even if observers were aware of the stimuli (Vadillo et al., 2016, 2020; Yaron et al., 2024).

At the same time, negative findings might also be driven by methodological issues. The same problem of post hoc trial exclusion might effectively lead to having a relatively low number of trials in which the unconscious effect is measured. This reduces the signal-to-noise ratio, and thereby the power (Baker et al., 2021), making it less likely to detect genuine unconscious effects, which are typically weak (Mudrik & Deouell, 2022). Another problem is that in attempting to prevent conscious perception of stimuli, researchers might employ too strong suppression methods. This can reduce the signal evoked by the stimulus to a degree that it cannot be sufficiently processed, leading to an underestimation of the true potential of unconscious effects (Michel, 2023; Reingold & Merikle, 1988; Stockart et al., 2025; Wentura et al., 2024). This, in turn, can lead to an overestimation of the functions of consciousness, as they are typically implied by lack of an unconscious effect (Lange et al., 2011; Sackur & Dehaene, 2009). This concern is based on the assumption that stimuli of weaker intensity will evoke a weaker unconscious effect, an assumption that has repeatedly been demonstrated in the literature (Cohen et al., 2024; Cul et al., 2007; Mashour et al., 2020), and is also compatible with some theories of consciousness, like the Global Neuronal Workspace (Dehaene, 2001).

Ideally, then, one would want a paradigm that (a) minimizes conscious contamination, and hence the need to exclude trials or participants; and (b) maximizes the signal elicited by the unconscious stimulus. A possible way to achieve these goals is using a calibration method (Shanks, 2017; see also Stockart et al., 2025), aimed at finding the highest stimulus intensity per participant where the stimulus is not consciously perceived.

**Fig. 1:**
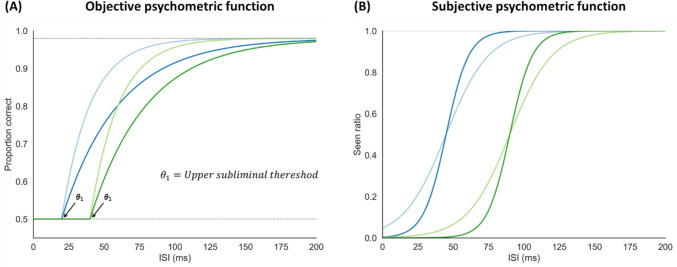
Illustration of the objective and subjective psychometric functions. **(A)** The objective psychometric function models the proportion correct across ISI values. Each curve represents a different combination of ${\theta }_{1}$ (upper subliminal threshold) and ${\theta }_{2}$ (improvement rate after the threshold is passed). The *gray horizontal lines* mark the lower (0.5, chance performance) and the upper asymptotes (0.98; defined by a fixed lapse rate). **(B)** The subjective psychometric function models the proportion of trials reported as “seen” across ISI values; Each curve represents a different combination of ${\theta }$ (the function’s midpoint) and σ (the spread of the function). Different colors correspond to psychometric curves with different parameter combinations

In common psychophysical calibration methods, perception is typically modeled using a continuous psychometric function, where participants’ performance in some task about the stimulus (e.g., the objective measure in the context of unconscious processing) is modeled as a function of stimulus intensity (Bass et al., 2010; Macmillan & Creelman, 2004). Under this framework, any non-zero stimulus intensity produces some sensitivity to the presence​ of the stimulus or to its features, such that chance-level performance is theoretically possible only when stimulus intensity equals zero. Therefore, this model is not adequate for unconscious processing research, where the goal is to find a stimulus intensity that allows its presentation (hence, its intensity is necessarily above zero) while resulting in chance-level performance. Hence, a crucial assumption for finding unconscious processing, if it exists, is that there are stimulus intensities in which we are not consciously aware of the stimulus (defined as chance level performance in the objective measure), but still might produce enough signal to allow some information to be processed unconsciously. Rouder and Morey (2009) proposed a revised framework to bridge this gap; They defined a *task threshold*, which is the highest stimulus intensity for which objective performance is indistinguishable from chance. Under this framework, the psychometric function is divided into two parts (see Fig. 1A): stimulus intensities below the task threshold are modeled as having zero sensitivity, while intensities above the threshold follow the standard continuous psychometric function. Here, we refer to the task threshold as the ‘upper subliminal threshold’.

Accordingly, when researchers of unconscious processing use calibration, they typically target this upper subliminal threshold, finding the intensity that allows effective suppression at the strongest signal possible. This can potentially solve the above-mentioned problems: if the calibration procedure can accurately estimate the threshold, then conscious contamination would be minimized and, accordingly, the issue of trial and participant selection would be mitigated. No less importantly, the evoked signal would be maximized to allow for unconscious processes to take place, if it exists.

Indeed, several studies have used calibration to determine upper subliminal thresholds per participant (e.g., Bernstein et al., 1989; Blakemore et al., 2017; Eimer & Schlaghecken, 2002; Hung et al., 2016; Kiepe & Hesselmann, 2024; Lamy et al., 2008). Many of them have used the adaptive staircase method (Dixon & Mood, 1948; Levitt, 1971) that systematically adjusts stimulus intensities based on participants' responses: in each trial, the stimulus is presented at a given intensity, and participants are asked to perform some task on it (e.g., report whether it was presented upright or inverted). If participants respond correctly, the intensity of the stimulus is decreased by a certain amount (henceforth ‘step size’); if they respond incorrectly, it is increased by it. The calibration ends when reaching a predefined number of reversals (i.e., the turnaround points where performance fluctuates between being correct and incorrect), or trials. The resulting threshold is then determined by a predefined function. For example, by averaging the intensity at the reversal points (Peel and colleagues, 2018), choosing the final contrast level reached (Hung et al., 2023) or the second-highest level reached in the final batch of trials (Biderman & Mudrik, 2018; Faivre et al., 2019; Tal & Mudrik, 2024). Other studies relied on subjective reports rather than an objective task to estimate the threshold, and adjusted the contrast based on participants’ reports of having perceived the stimulus or not (e.g., Benthien & Hesselmann, 2021; Handschack et al., 2022, 2023; Hesselmann et al., 2016, 2018; Rothkirch & Hesselmann, 2018).

However, the efficiency, accuracy, and reliability of these calibration methods for finding upper subliminal thresholds have been questioned (Faes et al., 2007; Leek, 2001) for two main reasons. First, these methods were all developed for estimating thresholds that are around the midpoint area of the psychometric function (Dixon & Mood, 1948; Faes et al., 2007; García-Pérez, 1998; Kaernbach, 1991; Levitt, 1971; Taylor & Creelman, 1967; Watson & Pelli, 1983), rather than the lowest part of the function (i.e., ≤ task threshold in the Rouder & Morey framework), where performance is at chance, which is the one relevant for studying unconscious processing. Within the latter range, binomial noise is at its maximum. This renders participants’ responses less informative, as they are based on guesses (Faes et al., 2007; Leek, 2001). Thus, relying on such responses to adjust the estimated upper subliminal threshold would lead to less reliable threshold estimations, compared to when targeting above chance performance (i.e., the midpoint area). Second, chance level performance is not uniquely associated with the upper subliminal threshold, but also with any subliminal intensity below it (see again Fig. 1A). Hence, even when a candidate method successfully yields a subliminal intensity level, it may underestimate the upper subliminal threshold, leading researchers to choose a weaker stimulus signal and thereby decreasing the chances of finding an effect. This raises the concern that current calibration methods might not be optimal for studying unconscious processes.

In this paper, we first demonstrate that this concern is indeed valid by showing that current calibration methods fail to identify the upper subliminal threshold reliably. Then, we introduce a novel method, specifically designed for the study of unconscious processing. We use both simulations and experiments to evaluate our method, demonstrating its effectiveness in setting optimal stimulus intensities for unconscious processing studies, compared to existing approaches.

## General simulations framework

In this section, we describe the process by which we simulated data for estimating upper subliminal thresholds (all code used to conduct these simulations is available at https://github.com/EdenElbaz10/STEP-calibration). To provide a quick overview, we first ran an empirical experiment to estimate the characteristics of psychometric curves in a masking experiment, which is commonly used to study unconscious processing. Specifically, we used metacontrast masking (Remole, 1985; Werner, 1935). Then, we treated these curves as the true psychometric curves underlying the performance of the virtual participants to generate trial-by-trial data in our simulations. In addition, we extracted the “true” upper subliminal threshold of each virtual participant from these curves to test different calibration methods, asking how effective they are in finding these thresholds. Readers who are less interested in the methodological details can skip to the “Simulation results” section below.

### Obtaining realistic psychometric functions

To provide a realistic data-generating process in our simulations, we obtained the psychometric curves by first collecting empirical data from forty healthy volunteers (30 females, three left-handed; aged 19–30, M = 23.4, SD = 3.14),^1^ who participated in the experiment in exchange for course credit or payment (~ $10). This use of real data allowed us to model more realistic psychometric functions that represent participants’ performance across different stimulus levels. Specifically, we simulated the performance of ‘virtual participants’ based on a psychometric analysis of the performance of actual participants, to extract the parameters of common psychometric curves as well as the associated estimated upper subliminal threshold. This threshold was taken as the ‘ground truth’ for the virtual participants, allowing us to test how well the different calibration methods manage to estimate it.

The experiment used a metacontrast masking paradigm, where the critical stimulus was either a left or a right arrow, followed by a blank screen and then a larger bidirectional arrow (Vorberg et al., 2003; henceforth the mask). To capture the entire perceptual spectrum of the critical stimulus, we used the common solution of Method Of Constant Stimuli (MOCS; Kingdom & Prins, 2016). In this method, a set of predetermined stimulus intensities is presented multiple times in random order. These intensities are chosen to evoke objective performance on the critical stimulus, ranging from chance level (50%) to near perfect (~ 100%). Following Di Lollo and colleagues (2004), we operationalized the calibration-estimated stimulus intensity as the inter-stimulus-interval (ISI) between the critical stimulus and the mask (0, 8.33, 16.67, 25, 33.33, 58.33, 100, 141.70, 200 ms; 64 trials each, intermixed; for further details, see Methods section below, where we report Experiment 1 that was identical in all parameters to this one, but used STEP calibration rather than the MOCS).

At the end of each trial, participants rated the visibility of the critical stimulus (i.e., the arrow) using the Perceptual Awareness Scale (PAS; Ramsøy & Overgaard, 2004), ranging from 0 (“I saw nothing”), 1 (“I had a vague glimpse, I don’t know what it was”), 2 (“I saw the arrow almost clearly”), to 3 (“I saw the arrow clearly”), and then performed an objective discrimination task (2AFC), by determining if the arrow was pointing rightwards or leftwards.

For all the participants in the experiment we ran, we used Bayesian hierarchical fitting to fit two psychometric functions, representing performance and ratings in the objective and subjective measures, respectively, across ISIs. We chose the hierarchical approach to overcome participant-level noise in estimating the threshold, while leveraging shared information across participants (Katahira, 2016). The objective psychometric function was modeled using a modified Weibull function (see Eq. (1) and Fig. 1A), adapted from (Rouder & Morey, 2009).

1 $$f\left(ISI;{\theta }_{1}, {\theta }_{2}\right)=\left\{\begin{array}{cc}0.5+(0.5- \lambda )\cdot \left(1-{{e}^{-}}^{\left(\frac{ISI-{\theta }_{1}}{{\theta }_{2}}\right)}\right),& x>{\theta }_{1}\\ 0.5,& x\le {\theta }_{1}\end{array}\right.$$

where ${\theta }_{1}$ denotes the upper subliminal threshold (the point at which performance begins to rise above chance level) and ${\theta }_{2}$ denotes the rate of performance improvement after the threshold is passed. The lower asymptote was set at 0.5 (chance level performance), and the upper asymptote was set at 0.5 + (0.5—$\lambda$). Specifically, $\lambda$ was defined in order to account for lapses of attention or unintended response errors that may occur, and was set to 0.02, allowing the function to vary within the range ∈ [0.5, 0.98] (see Faes et al., 2007; García-Pérez, 2000; García-Pérez, 1998 for an explanation of this range). The objective threshold ${\theta }_{1}$ was taken as the ‘ground truth’ for participants’ threshold, and accordingly the different calibration methods were tested for their accuracy in estimating ${\theta }_{1}$.

To model subjective responses, we used a logistic cumulative distribution function (see Eq. (2) and Fig. 1B). To fit the parameters of this function to participants' responses, we transformed the PAS ratings into a binary scale: trials with a PAS rating of 0 were categorized as ‘not seen’, whereas trials with PAS ratings higher than 0 were categorized as ‘seen’ (e.g., Handschack et al., 2022; Soto et al., 2011; Stockart et al., 2025; Trübutschek et al., 2017):

2 $$f\left(ISI;\theta ,\upsigma \right)=\frac{1}{1+ {e}^{ \frac{\theta - ISI}{\upsigma }}}$$

where ${\theta }$ denotes the function’s midpoint and σ determines the spread of the function (Treutwein & Strasburger, 1999).

The priors for the Bayesian model for the objective (${\theta }_{1}$, ${\theta }_{2})$ and subjective psychometric functions ($\theta ,\upsigma$) were modeled using a truncated normal distribution constrained to positive values. To ensure that the model accurately captured individual differences in model parameters while avoiding overly restrictive constraints, we specified broad, mildly informative normal priors for the ISI threshold of the objective psychometric functions (${\theta }_{1}$) as $\mu =25$ and $\sigma =5$ (the value found to be most effective in suppressing the stimulus in Peremen & Lamy, 2014). The same priors as ${\theta }_{1}$ were used for the remaining parameters, given that our results were not sensitive to prior specification (see Supplementary Materials 1 for sensitivity analyses).

Each simulation included 10,000 iterations (i.e., 10,000 virtual participants whose true psychometric curves were identical to the psychometric functions estimated for the actual 40 participants who underwent the MOCS experiment, as explained in the previous section). Thus, each psychometric function obtained in the experiment was replicated 250 times, such that each virtual participant had a designated psychometric function, reflecting the population variability as estimated from empirical data. Then, the responses of each participant for a given ISI were generated by first randomly selecting a number from a uniform distribution ranging from 0 to 1; if this random number was smaller than the true proportion correct for this ISI value according to the participant's psychometric function, the simulated response was classified as correct. Conversely, if the random number was larger than the proportion correct value, the simulated response was classified as incorrect. Hence, the expected performance of each virtual participant in each ISI was determined according to its psychometric function. The true upper subliminal threshold for these participants was also derived from their true psychometric functions, using Eq. (1) above.

### Testing calibration methods used in unconscious processing studies

**Fig. 2:**
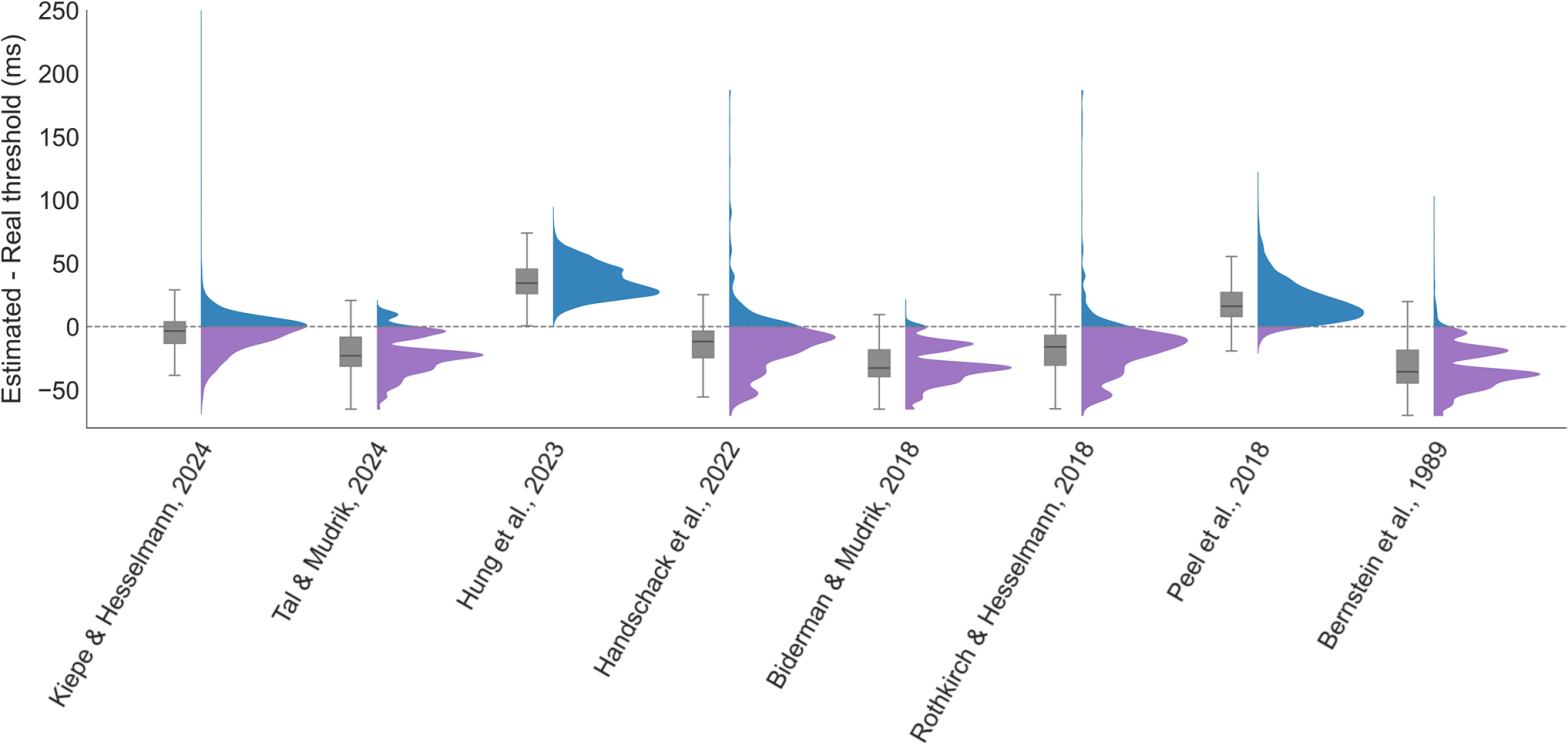
Accuracy analysis of calibration procedures used in eight unconscious processing studies. Accuracy was assessed using simulations (10,000 virtual participants per method) and is presented as the threshold estimation error, the difference between the estimated and true upper subliminal threshold. The distributions thus aggregate the results across these simulations, showing how frequently each deviation from the real upper subliminal threshold occurred across all iterations. Positive values indicate overestimation (*blue*), negative values indicate underestimation (*purple*). Accordingly, values closer to zero reflect greater accuracy, with the dashed line at zero indicating perfect threshold estimation. The *x*-axis lists study labels and the *y*-axis indicates the density over threshold estimation error. Hence, the distribution for each study provides a full description of error values, including the central tendency and variability, thereby reflecting both the reliability (narrowness of distributions) and bias (systematic over- or underestimation) of each calibration approach. Overlaid *gray boxplots* represent the median, interquartile range, and overall spread. Each study label corresponds to a specific calibration method as reported in the original publication: Staircase with fixed step size (Bernstein et al., 1989; Biderman & Mudrik, 2018; Tal & Mudrik, 2024), adaptive step size (Handschack et al., 2022; Hung et al., 2023; Peel et al., 2018; Rothkirch & Hesselmann, 2018), or QUEST (Kiepe & Hesselmann, 2024).These methods vary in step size, stopping rule, awareness measure (objective/subjective), and threshold calculation. See Supplementary Table S1 for a detailed summary of each method

The aim of all simulations was to assess the accuracy of different calibration methods in detecting the true upper subliminal threshold per virtual participant (${\theta }_{1}$). In each iteration, we calculated the difference between the threshold estimated by each method and ${\theta }_{1}$. We then aggregated these differences over all iterations to generate the full distribution for each method (Fig. 2).

Specifically, we focused on calibration methods used in a collection of eight studies in the field (Fig. 2; note that although these studies were gathered through an extensive literature search, we did not conduct a systematic review procedure (Page et al., 2021). Thus, this should not be taken as an exhaustive sample of studies using calibration methods in the field). For each such study, we implemented the reported calibration procedure based on the published descriptions and ran it on our virtual participants with the initial ISI set to 200 ms.

As some of these studies manipulated stimulus intensity via other parameters rather than the ISI that was used here (e.g., contrast, duration), a transformation was performed to make the manipulated parameter values comparable (see Supplementary Materials 2 for details, as well as more information on the specific method used by each study).

## Simulation results

All tested calibration methods used in the examined studies were surprisingly unreliable at estimating upper subliminal thresholds: in most cases, the simulations were found to be relatively far from the actual threshold, and even when the result was close (Kiepe & Hesselmann, 2024; Rothkirch & Hesselmann, 2018), the variability was still quite large, such that for some participants, the error was larger than 50 ms (which is equivalent to average error of 79.08% between the difference in expected performance and chance level performance in these cases). One could claim, however, that this limited performance might stem from the relatively small number of trials used (20–100). To test this claim, we ran an additional control analysis, increasing the number of trials to 156 (matching our proposed solution), and the results did not improve (see Supplementary Materials 3). These limitations highlight the need for a calibration method specifically designed for unconscious processing studies, such that it can reliably identify upper subliminal stimulus intensities. This would allow researchers to examine the existence and scope of unconscious processing more adequately.

## The proposed solution: Subliminal Threshold Estimation Procedure (STEP)

Our proposal is based on the 1-up 1-down staircase method, yet with three changes that render it especially suited for finding the upper subliminal threshold. The first pertains to the way the threshold is calculated, taking into account the number of incorrect responses, the second also integrates the subjective measure, and the third defines an upper boundary for the threshold, to prevent overestimation and facilitate convergence on the correct threshold. To make the results more robust to random noise in responses, the method is conducted in two calibration runs, such that the final threshold is the average of the two obtained thresholds to increase reliability by minimizing anomalies or errors (Kontsevich & Tyler, 1999). Below, we describe the three key changes introduced in STEP in greater detail. Again, less interested readers can skip to the section “Testing the proposed method”, to compare STEP's performance with that of existing methods. Notably, all code needed to run this calibration procedure is available at https://github.com/EdenElbaz10/STEP-calibration/blob/main/calibration_methods/STEP.R).

### Cumulative-weighted incorrect responses (CWIR)

Staircase calibration methods typically target reversals, when participants’ responses fluctuate between being correct and incorrect, in two ways: defining the stopping rule (e.g., when reaching a certain number of reversals), and/or determining the calibrated threshold, by averaging over the mean intensity of the stimulus during these reversals (Blakemore et al., 2017; García-Pérez, 1998; Maffei et al., 2024; Peel et al., 2018). However, it is unclear whether it is effective to rely on reversals as indicators of chance level performance. To illustrate, consider the probability of obtaining a reversal when participants are at chance (50% correct responses in 2AFC task), which is equal to 0.5 $\left(p\left(correct, incorrect\right) \right| p\left(incorrect, correct\right)=0.5 x 0.5+0.5 x 0.5$). This probability is very close to the probability of obtaining a reversal when participants’ performance is above chance level (e.g., 60% correct responses); then, the probability equals 0.48 $\left(p\left(correct, incorrect\right) \right| p\left(incorrect, correct\right)=0.6 x 0.4+0.4 x 0.6$). Hence, in this case, a reversal cannot reliably distinguish between above-chance and chance-level performance, resulting in inaccurate calibrated thresholds.

Therefore, we replaced the reversal-based stopping rule with a requirement for sequences of *consecutive incorrect responses*, which provides a clearer differentiation between chance and above-chance performance levels. Here, the probability of obtaining two consecutive incorrect responses at chance level is 0.25 $(p\left(incorrect, incorrect\right)=0.5 x 0.5$) while at a performance level of 60%, the probability is 0.16 $(p\left(incorrect, incorrect\right)=0.4 x 0.4$).

Additionally, instead of simply counting incorrect responses, the proposed method assigns increasing weights to longer consecutive incorrect responses, such that each additional consecutive incorrect response is given a weight that is incremented by one (see Eq. 3). As a result of this non-linear increase, longer sequences have a greater impact.

3 $$CWIR\left(t\right)=\begin{array}{cc}0,&Correct(t)=1\\1+CWIR\left(t-1\right),&Correct(t)=0\end{array}$$

where $t$ denotes the trial number, *Correct* marks if the participant was correct in trial number t, and $CWIR\left(t\right)$ is defined recursively based on previous trials.

Each sequence contributes to the final threshold in proportion to its CWIR value. For a sequence of *k* consecutive incorrect trials, the CWIR is calculated as:

4 $$CWI{R}_{k}= \frac{k (k+1)}{2}$$

At the end of the calibration, the estimated threshold ($\widehat{\theta }$) is calculated as the weighted average of the means of the stimulus intensity for which these sequences occurred, with each mean intensity being weighted according to its sequence cumulative weight (CWIR):

5 $$\widehat{\theta }= \frac{{\sum }_{i=1}^{n}CWI{R}_{i}*{\overline{s} }_{i}}{{\sum }_{i=1}^{n}CWI{R}_{i}}$$

Here, ${\overline{s} }_{i}$ is the average stimulus intensity within the *i*^th^ sequence of consecutive incorrect responses, $CWI{R}_{i}$ is the total CWIR weight for sequence number *i*, and *n* is the number of such sequences during each calibration run.

### Integration with a subjective measure

The second change we introduce in the proposed calibration procedure is the inclusion of subjective measures (here, the PAS) alongside an objective measure of awareness. Specifically, as described above, we convert PAS responses into a binary scale, such that PAS = 0 is defined as ‘not seen’ and PAS = 1, 2, 3 as ‘seen’. Consequently, each trial can result in one of four outcomes (seen & incorrect, seen & correct, not seen & incorrect, not seen & correct). For ‘seen & incorrect’ responses, participants may have been subjectively aware of the stimulus but still provided an incorrect response due to momentary lapses of attention. Therefore, in such a case, we do not increase stimulus intensity despite the incorrect response. In all other cases, the subjective measure does not affect the intensity adjustment procedure.

Importantly, this rule is only applied at the beginning of the calibration (before reaching the minimal step size, see below), where step size is larger and accordingly has a high effect on the resulting calibrated threshold. Not following the rule throughout the rest of the calibration is aimed at avoiding a scenario where participants never rate visibility as 0 (e.g., due to having a higher criterion; Eriksen, 1960; Michel, 2023), potentially leading to an underestimation of the threshold (because the intensity of the stimulus is never decreased under this scenario). We further note that the inclusion of the subjective measure is not mandatory in STEP. Accordingly, we provide an analysis of the performance of a “subjective measure-free” variant of STEP, such that researchers who prefer not to use this measure would still be able to employ our method (see Supplementary Materials 4).

### Upper boundary

Finally, to improve efficiency and prevent overestimating the threshold, we introduce a third modification: a dynamic upper boundary that limits the range in which the estimated threshold lies. This prevents stimulus intensity from sharply increasing during calibration due to noisy responses, biasing the calibration away from the target threshold. At the beginning of the calibration, the upper boundary is set at the initial stimulus intensity (i.e., the maximum examined intensity). Then, in each trial, the responses from all trials with stimulus intensity within the range of the current intensity and the boundary are selected. To test whether the upper boundary should update, we use a one-tailed binomial test to estimate whether the proportion of correct responses of those trials is significantly different from chance level performance (0.5). A significant result is taken as evidence for the current upper boundary being too liberal, leading to an update of the boundary. To determine significance while compensating for multiple comparisons, we adjust the criterion for significance, $\mathrm{alpha}=\frac{0.05}{CWIR+ 1}$ (1 is added to prevent division by zero). The division by CWIR is aimed at achieving greater correction as one approaches the true threshold (since the likelihood of finding a large magnitude of cumulative weighted incorrect responses is lower the farther one is from chance). When we have more incorrect responses, the probability of finding above-chance performance in the binomial test decreases. Thus, we decrease the alpha to make the test harder to pass, thereby preventing false positives. Then, the new boundary is defined as a function of the average of the previous boundary and the stimulus intensity for which significant performance was found. This average is weighted based on the proportion of trials in which participants indicated seeing the prime out of all trials whose stimulus intensity lies between the boundary and the stimulus intensity in the current trial (seen ratio):

6 $$newBoundary = \left(1-seenRatio\right)\cdot Boundary+ seenRatio\cdot current Intensity$$

The rationale for weighting the boundary by the seen ratio is that the new boundary should reflect the degree of subjective awareness; if participants saw many of the stimuli in the trials whose stimulus intensity was between the current intensity and the current boundary, we would like the updated boundary to get much closer to the current intensity. Accordingly, we assign the current boundary a smaller weight ($1-seenRatio$, as $seenRatio$ is high), and the current intensity a larger weight ($seenRatio$). Alternatively, if participants did not see many trials, the updated boundary will remain relatively close to the current boundary, for the same reason (note that this boundary modification works well even when not accounting for the seen ratio, in case researchers choose not to measure subjective awareness; see Supplementary Materials 4).

With this adjustment, the boundary will move closer to the lowest stimulus intensity for which participants are still above chance, thereby narrowing the range of stimulus intensities to those most relevant for calibration. As a result, it could reduce threshold overestimation and improve the efficiency of the process.

## Testing the proposed method

To evaluate the accuracy of the STEP method, we compared its performance with several well-established calibration procedures commonly used in psychophysical research, including the staircase procedure (Levitt, 1971), Parameter Estimation by Sequential Testing (PEST; Taylor & Creelman, 1967), QUEST (Watson & Pelli, 1983), QUEST + (Watson, 2017) (with some modifications; see Supplementary Materials 5), and the Accelerated Stochastic Approximation method (ASA; Faes et al., 2007; Kesten, 1958). Simulations were conducted using the same General Simulation Framework described above, with several adjustments to these methods. These, pertained to (a) the number of trials, which was equated between methods; (b) the definition of the stopping rule; and (c) the estimated threshold. Factors (b) and (c) were determined based on each method (see Supplementary Materials 5 for details).

## Evaluation metrics

We used three complementary metrics for assessing the performance of the different calibration methods in estimating the upper subliminal threshold. Each provides a somewhat different estimation of the effectiveness of the different methods (and suffers from different biases), rendering their results complementary when assessing them.

The root mean square error (RMSE) was calculated as the deviation between the estimated threshold (${\widehat{\theta }}_{1i}$) and the true threshold $({\theta }_{1i})$:

7 $$\mathrm{RMSE}=\sqrt{\frac{1}{N} {\sum }_{i=1}^{N}{\left({\widehat{\theta }}_{1i}-{\theta }_{1i}\right)}^{2}}$$

where RMSE quantifies the deviation between the estimated (${\widehat{\theta }}_{1i})$ and the true thresholds $({\theta }_{1i})$ divided by the number of participants ($N$).

The normalized root mean square error (NRMSE) was calculated as follows:

8 $$NRMSE=\sqrt{\frac{1}{N}{\sum }_{i=1}^{N}{\left(\frac{ {\widehat{\theta }}_{1i} - {\theta }_{1i}}{{\theta }_{1i}}\right)}^{2}}$$

where NRMSE quantifies the relative threshold estimation errors by normalizing these errors by the true threshold values. For some psychometric functions, RMSE might be less suitable: RMSE may introduce bias if the absolute threshold is correlated with the error: a 5-ms error might have a different meaning when the true threshold is 5 ms than when it is 80 ms, something RMSE does not capture, but NRMSE does (since the error is proportional to the true threshold). Moreover, when the true threshold is close to zero, negative deviations are truncated in the RMSE calculation (since the estimated thresholds cannot be less than 0), creating an asymmetry in the error range in which mostly overestimations are possible (leading to floor effect). NRMSE adjusts for it by expressing error relative to the true threshold, thereby better capturing performance across the full range of possible values.

Finally, the Proportion Correct RMSE (PRMSE) was calculated as the RMSE between participants' probability of being correct at their estimated threshold and $P\left({\widehat{\theta }}_{1}\right)$ and chance level probability (50%):

9 $$\mathrm{PRMSE}=\sqrt{\frac{1}{N} {\sum }_{i=1}^{N}{\left( P({\widehat{\theta }}_{1i}) -0.5\right)}^{2}}$$

This metric specifically evaluates the impact of errors in the estimated thresholds, by quantifying the deviation of the resulting true performance in the objective measure from chance-level performance, which is crucial for unconscious processing paradigms. The RMSE and NRMSE are symmetrical metrics that only indicate the deviation of the estimated threshold from the true threshold value, regardless of whether the deviation is an underestimation or an overestimation. The PRMSE is asymmetric because it penalizes only estimated values that are above the true upper subliminal threshold. Hence, higher PRMSE values indicate a higher level of conscious contamination. The asymmetry of PRMSE reflects the asymmetry in the potential impact of errors in estimating the upper subliminal threshold. While underestimation might increase type 2 error, overestimation will lead to inflated type 1 error (falsely inferring unconscious processing, in the face of conscious contamination). For example, if two different calibration methods have the same RMSE and NRMSE but the PRMSE of one method is higher, this means that the deviation from the true threshold is the same in both methods, but in the method with the higher PRMSE, the threshold is more often overestimated, leading to above-chance performance. This would suggest that the second method is preferable, despite having the same RMSE and NRMSE scores.

**Fig. 3:**
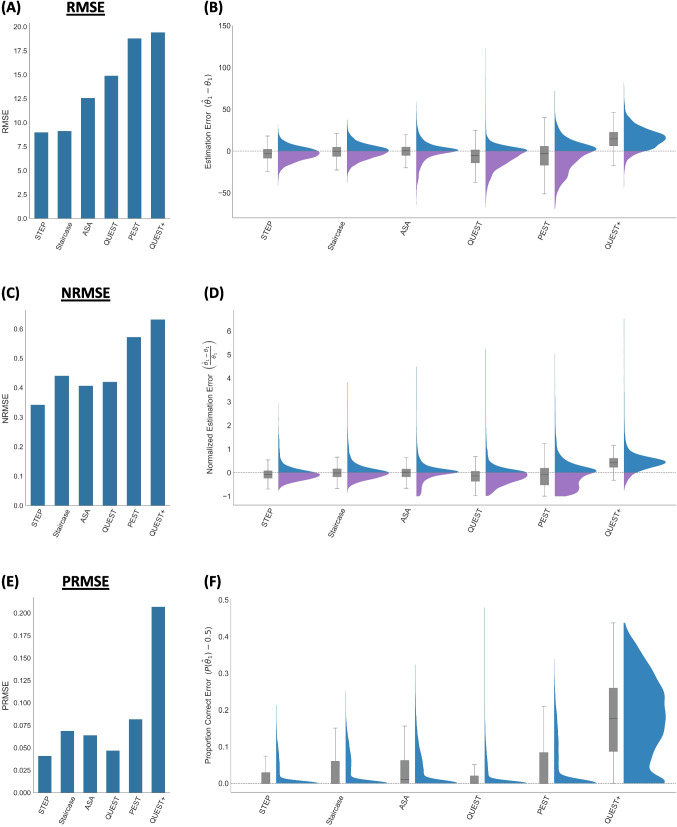
Accuracy of calibration procedures across three error metrics (lower values indicate better performance) and their corresponding distributions. *Top row:* Estimation error with** (A)** the RMSE values and **(B)** the difference between the estimated and true threshold (${\widehat{\theta }}_{1}$–${\theta }_{1}$). Positive values indicate overestimation (*blue*), negative values indicate underestimation (*purple*). Accordingly, values closer to zero reflect greater accuracy, with the dashed line at zero indicating perfect threshold estimation. The *x*-axis lists study labels, and the *y*-axis indicates the density over threshold estimation error. Hence, the distribution provides a full description of error values, including the central tendency and variability, thereby reflecting both the reliability (narrowness of distributions) and bias (systematic over- or underestimation) of each calibration approach. Overlaid *gray boxplots* represent the median, interquartile range, and overall spread; *Middle row*: relative error normalized by the true threshold with **(C)** NRMSE values and **(D)** normalized estimation error ((${\widehat{\theta }}_{1}$–${\theta }_{1}$)/${\theta }_{1}$). The same standards apply as in panel **(B)**; *Bottom row*: Proportion correct error with **(E)** PRMSE (deviation of predicted performance from chance level) and **(F)** the deviation of the resulting performance of the estimated threshold in the objective measure from chance-level performance ($P$(${\widehat{\theta }}_{1})$–0.5). The same standards apply as in panel **(B)**, though note that here, the difference cannot be negative since the minimal value of $P$(${\widehat{\theta }}_{1})$ is 0.5. Across all metrics, the STEP method consistently outperformed the staircase, ASA, QUEST, PEST, and QUEST + methods. For results including STEP without the subjective measure, see Supplementary Materials 4

Our simulations suggest that when all metrics are considered, the STEP method outperformed all others (Fig. 3): it yielded the lowest RMSE (8.99). Although the RMSE values for STEP and the staircase method were comparable, when threshold errors were normalized by the true upper subliminal threshold, STEP revealed a clearer advantage (NRMSE = 0.34), relative to all other methods. This further confirms the improved generalizability and robustness of the STEP method across different threshold ranges. Finally, in terms of how closely the estimated thresholds aligned with chance-level performance (50%), STEP demonstrated the smallest deviation (PRMSE = 0.04), suggesting that it most accurately identified perceptual boundaries that are truly subliminal (since conscious contamination is minimized), allowing researchers to test whether unconscious processing takes place, and to what extent. Taken together, these results demonstrate that the STEP method is superior compared to traditional calibration procedures across all three accuracy metrics, providing more precise and theoretically meaningful threshold estimates for unconscious processing studies.

## Testing STEP empirically

The simulations we ran demonstrated the superiority of STEP over other calibration methods. But to directly examine its advantages, we ran three experiments testing whether it indeed yields less trial and participant exclusions compared with an experiment that does not include a calibration, and if it results in non-significant performance in the objective measure at the group level. Here, we used a priming procedure, where the critical stimulus is rendered invisible and is either congruent or incongruent with a visible target stimulus, on which participants perform a task (Kinoshita & Lupker, 2003). Better performance in similar prime-target trials (i.e., priming) is held as evidence for prime processing, and chance-performance in the objective measure is held as evidence for it being effectively suppressed from awareness (see the "Standard Dissociation" paradigm in Schmidt & Vorberg, 2006).

In the first two experiments, participants completed the calibration procedure on the prime-mask ISI, followed by a meta-contrast masked motor priming task (Vorberg et al., 2003). The first experiment compared two groups: one in which the estimated threshold was used, and the other where a fixed value was used. Experiment 2 was aimed at replicating the findings of the calibration group from Experiment 1 in a preregistered study (https://osf.io/vx8n9/). In Experiment 3, we examined the generalizability of the STEP method to manipulating the contrast instead of the ISI, by testing its ability to estimate upper subliminal thresholds in an affective priming task (Jiang et al., 2013).

## Methods

### Participants

A total of 89 participants took part in the study in exchange for course credit or payment (~ $10): Experiment 1 included 46 participants who were divided into a calibration group (15 females and five males, 19 right-handed; age M = 23.9 years, SD = 3.75) and a control group (15 females and 11 males, 23 right-handed; age M = 24.8 years, SD = 4.14). Notably, six participants were excluded from the control group due to above-chance performance, such that eventually we had 20 participants in each group. Experiment 2 included 22 participants (18 females and four males; 21 right-handed; age: M = 24.62 years, SD = 3.13), out of which two were excluded. Experiment 3 included 21 participants (17 females, four males; 19 right-handed; age: M = 22.95 years, SD = 1.24, one excluded). Exclusion criteria also included (1) failure to complete all trials in the calibration procedure, (2) low performance in the main task (i.e., accuracy lower than 70% in the target discrimination question), suggesting that they are unable to follow the instructions, and (3) rated less than 200 trials as unaware, which jeopardizes the sensitivity of the objective measure (Yaron et al., 2024) or less than 50 trials in each experimental cell after trial exclusion. These criteria were determined before analyzing the main effects, and no additional outliers were removed based on participants' results. Notably, the pattern of the results did not depend on participant exclusion(see Supplementary Materials 6).

### Apparatus and stimuli

Stimuli were presented on a ViewPixx LCD monitor (1920 × 1080 pixels, 120-Hz refresh rate) using PsychoPy. Participants sat in a dark room with their heads stabilized using a chin rest positioned 60 cm from the screen. In Experiments 1 and 2, the prime was a left/right-pointing arrow (0.8° × 1.86°). The target was a larger arrow with the center cut out, to ensure that it does not occlude the area where the prime was presented. In the calibration, the mask was a larger bidirectional arrow (1.09° × 3.47°). In the main task, the mask also served as a target, which was larger (unidirectional arrow, 1.09° × 2.91°). Following Vorberg et al. (2003), prime and target appeared randomly either 1.38° above or below a fixation cross. All stimuli were black on a white background. In Experiment 3, stimuli were 72 facial expressions (6° × 4.8°) taken from the Karolinska Directed Emotional Faces collection (Goeleven et al., 2008), including 36 happy and 36 fearful expressions. All faces were presented at the center of the screen as frontal, color images framed by a gray oval (RGB: 128, 128, 128), to remove the hair and any additional features (e.g., earrings). The faces were validated and equated for low-level features (see Supplementary Materials 7).

**Fig. 4:**
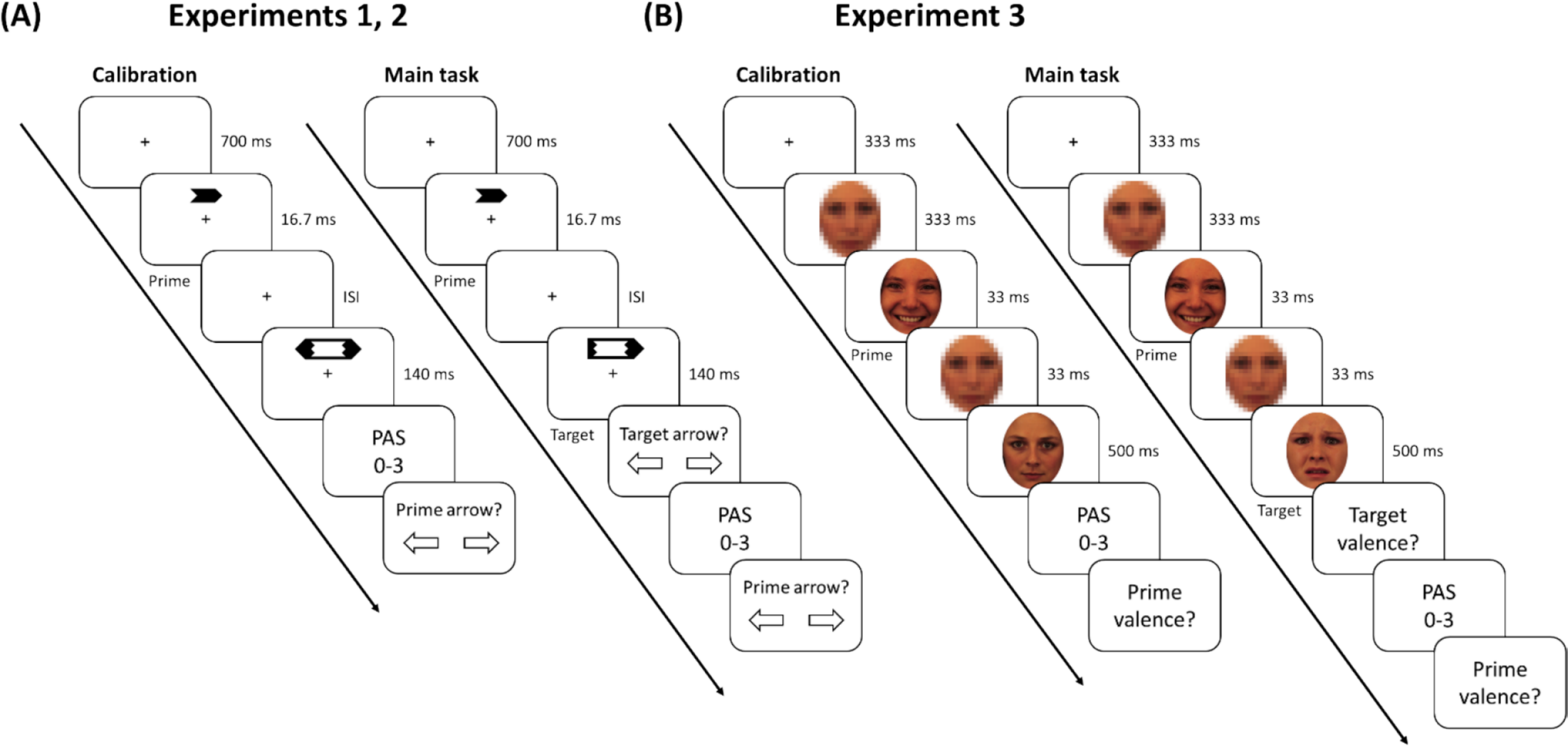
Experimental procedure for calibration phases and main tasks **(A)** Experiments 1 and 2: in both parts, a fixation cross was displayed for 700 ms, followed by the prime arrow for 16.7 ms, and then a fixation screen (ISI). Next, the target was presented for 140 ms. **(B)** Experiment 3: a fixation cross was presented for 333 ms, followed by a prime (positive or negative facial expression) for 33 ms, and a second mask (33 ms). Then, a target (a neutral facial expression in the calibration phase and a positive or negative facial expression in the main task) was presented for 500 ms. Across all main tasks, participants were instructed to classify the target. Additionally, during both phases, participants completed subjective (PAS) and objective (2AFC) awareness questions at the end of each trial

The masks used in both the calibration and the main task were created as pixelated versions of neutral facial images of twelve additional identities from the same stimulus set; Each image was resized to a lower resolution (pixel size: 20 × 20) using bilinear interpolation and then rescaled to its original size using nearest-neighbor interpolation, producing a pixelated appearance (see Fig. 4). This process was implemented in Python using the Pillow library. These scrambled masks served as primes in catch trials.

Primes and targets were pseudo-randomly assigned for each participant, such that 24 images served as primes, and the rest as targets. Individual identities that appeared in both the “fearful” and “happy” expression pools were consistently assigned the same role, with both expressions from that identity designated either as primes or as targets. Gender and emotional expressions were balanced across prime and target sets.

In the calibration phase, the primes consisted of happy/fearful faces that later served as target in the main task (to prevent participants from being consciously exposed to the prime images in the main task). The targets were the twelve neutral face images used to create scrambled masks.

### Procedure

#### Experiments 1 & 2

##### Calibration

Participants first completed ten practice trials. Each trial started with a fixation cross appearing at the center of the screen (700 ms), followed by the prime (left/right arrow;16.7 ms), and a blank screen for an adaptive ISI duration before the mask (a larger bidirectional arrow; 140 ms). Participants were asked to rate prime visibility using the four-point PAS and then discriminate the orientation of the prime (left/right) with the arrow keys. Responses to both questions were unspeeded.

As described above, the initial and maximal ISI was 200 ms and was adjusted according to the participant’s responses following the STEP rules. Due to the screen’s refresh rate, the original step size was 32 ms (4 frames). It decreased by 8 ms (one frame) rather than the 4 ms used in the simulations, until reaching the minimal step size (8 ms). To better mimic an effective step size of 4 ms (simulations), the ISI only changed on odd trials (increased if both responses were incorrect, decreased if both responses were correct, and remained the same for mixed responses), until the maximal or minimal ISI was reached (0 ms).

Akin to the MOCS experiment described above, the calibration procedure did not include a question about the target, to avoid potential task-difficulty artifacts associated with a dual-task paradigm (Pratte & Rouder, 2009). Additionally, a bi-directional arrow was used as a mask in the calibration to prevent contamination of the prime response by the direction of the target (Peremen & Lamy, 2014; Vermeiren & Cleeremans, 2012).

##### Main task

Following the calibration procedure, the main task began. For the calibration group, the ISI was set to the individually estimated threshold; for the control group (in Experiment 1 only), it was fixed at 25 ms, which is the closest value to the mean of the calibration group. Participants completed 17 practice trials with auditory feedback on their performance about the target, followed by 480 trials divided into ten blocks. Half of the trials were congruent (prime and target pointed to the same orientation), and half were incongruent (pointed to opposite orientations).Forty eight additional trials served as catch trials, with a black rectangle (0.7° × 1.2°) as a prime. Overall, there were 12 conditions combining three primes (left, right, catch) with two locations (up, down) and two targets (left, right) randomized within blocks.

The procedure was identical to the calibration block, except for the following changes: First, the target was an arrow oriented either left or right. Second, after the target presentation, participants were instructed to discriminate the orientation of the target (left/right), as fast as they can, using the arrow keys. Responses had to be provided within a 1000-ms time window from the target presentation. Responses slower than 1000 ms were followed by “Too slow” feedback. Then, participants answered the subjective (PAS) and objective (2AFC) questions as in the calibration procedure (unspeeded responses; Fig. 4). Note that unlike the calibration phase, in the main task participants responded to both the prime and the target, with the target being informative (directional arrow). This introduces the potential issue of attentional competition, as participants are likely to prioritize the response to the target over the prime, potentially leading to an underestimation of participants' actual discrimination ability (Vermeiren & Cleeremans, 2012). A potential way to avoid this issue is to test for consciousness in a separate block, yet this introduces additional problems; mainly, participants might change their strategies between the blocks, and their responses could also differ from the main experiment due to fatigue on the one hand and training on the other (Stockart et al., 2025). Thus, testing within trial was recently recommended as preferred over post-trial testing (Stockart et al., 2025). Here, we adopted this approach, notwithstanding its drawbacks.

The same procedure was used for Experiment 2, except for the number of trials, which was determined via power analysis to be 640 non-catch and 240 catch; 1000 iterations of a simulation of a one-tailed Fisher's exact test were conducted to calculate the number of trials needed to detect a difference in PAS ratings between catch and non-catch trials with small effect size (h = 0.2). The determined trial number was found to provide a power of 80%.

## Experiment 3

### Calibration

The procedure closely followed Experiment 2, with the following modifications: (a) Instead of adjusting the ISI, the contrast of the prime was manipulated. (b) The initial and maximal contrasts were set to 1. (c) The initial step size was set to 0.2 and was decreased by 0.05 until it reached the minimal step size (0.05). (d) The contrast of the prime was updated after each trial, unless participants reached the maximal or minimal contrast (0.1).

### Main task

First, participants completed 16 practice trials and received auditory feedback on their responses to the target question. The main task included 864 trials, 576 non-catch trials and 288 catch trials, divided into six blocks. Each block contained an equal number of congruent trials (prime and target from the same valence), incongruent trials (prime and target from different valences), and catch trials (masks appeared as primes). In total, the experiment included 12 conditions, combining three types of primes (happy, fearful, catch), two types of targets (happy, fearful) and two genders (female, male). In each block, for non-catch trials, each prime image appeared four times and each target appeared twice (randomly intermixed at different trials). Trial types were randomly assigned and evenly distributed across each block, with breaks between blocks.

Each trial included a fixation cross (333 ms), a mask (333 ms), a prime face (33 ms; at the individually calibrated contrast level), a mask (33 ms) and a target (500 ms). Then, the same responses were given as in Experiments 1 and 2, but here the target and prime discrimination tasks pertained to their valence using the arrow keys (button assignment was counterbalanced across participants; For a full trial sequence, see Fig. 4).

### Exclusion criteria

In all experiments, analyses included only subjectively unaware trials: to address the criterion problem, a one-tailed Fisher’s test was used to compare the distributions of PAS = 0 and PAS = 1 ratings between catch trials and non-catch trials. For participants with non-significant differences in PAS ratings between these conditions, trials with PAS ratings of 0 and 1 were counted as invisible, excluding trials rated as 2 or 3. In contrast, for participants where a significant difference was found, only PAS = 0 trials were considered invisible, excluding trials with PAS ratings higher than 0. Objective prime visibility was tested using a two-tailed *t* test against chance (50%).

In addition, for RT analyses, trials in which the RT for target classification was shorter than 150 ms or trials in which participants did not answer on time were excluded from all analyses. Additionally, trials in which the RT deviated by 3 SDs from the participant's mean RT in that condition were excluded. Trials where the response to the target question was incorrect were also excluded.

### Data analysis

In all experiments, objective awareness was tested using a two-sided *t* test at the group leve, for each groupl. Performances were compared with chance level (50%). As a complementary analysis, we performed a Bayesian *t* test, with the default prior. We interpreted a Bayes factor (BF_10_) < 0.33 as substantial support for the null hypothesis over the alternative hypothesis, 0.33 < BF_10_ < 3 as inconclusive results, and 3 < BF_10_ < 10 as moderate evidence for an effect.

Priming effects in all experiments were analyzed using a linear mixed-effects model (LMM) on target RTs, with congruency (congruent/incongruent) as a fixed effect and participant as a random effect at the trial level.

In Experiments 1 and 2, we included random intercepts and random slopes for congruency by participant, formally described as:

$$RT \sim Congruency+\left(Congruency \left|Participant\right.\right)$$

In Experiment 3, a random slope model resulted in a singular fit. Therefore, we only used random intercept:

$$RT \sim Congruency+\left(1\left|Participant\right.\right)$$

LMMs were implemented using the lmer function from the lme4 package in R, and *p* values were obtained via the lmerTest package with Satterthwaite’s approximation for denominator degrees of freedom.

We corrected for multiple comparisons for the priming effects using a Bonferroni correction. Specifically, since the priming effect was tested separately in four groups (Experiment 1 calibration, Experiment 1 control, Experiment 2, and Experiment 3), we applied a correction across these four RT analyses. Testing for objective performance was not included in this correction, for testing for objective awareness in a more conservative manner.

In Experiment 2, we complemented the priming analysis with a Bayesian LMM analysis with an ex-Gaussian distribution. This was only done for this experiment, as it was the only experiment for which we had meaningful priors. We used the results of Experiment 1 to determine the priors; There, the prior on the intercept was modeled with a normal distribution (M = 0.45, SD = 0.1), the condition effect was modeled with a Student’s *t*-distribution (DF = 3, M = 0, SD = 0.04 s), reflecting the effect found in Experiment 1 but centered at zero to ensure the prior did not bias results toward detecting an effect. Residual variance (sigma) was modeled with a Student’s t-distribution (DF = 3, M = 0.1, SD = 0.001), constrained to be ≥ 0.

Finally, given recent criticism against the common approach for testing unconscious priming (Meyen et al., 2022), we conducted a complementary post hoc analysis to test for an Indirect Task Advantage (ITA) by directly comparing sensitivity in the direct (objective) and indirect (priming) tasks. The SD value of true sensitivities across participants was q^2^ = 0.0025, to minimize the likelihood of false positives.

## Results

### Experiment 1

In the calibration group, the mean estimated ISI was 29.17 ms (SD = 17.58). In the main experiment, 95.09% (SD = 12.84) of trials were rated as subjectively invisible, compared to 87.88% (SD = 16.20) in the control group. χ^2^(1, 19,200) = 319.40, *p* <.001; including six excluded participants from the control group: M = 90.29%, SD = 14.87; χ^2^(1, 22,080) = 176.76, *p* <.001), indicating that the calibration procedure yields very few trials that need to be excluded (less than 5%).

The calibration was effective in reducing participant exclusion: no participant showed above chance performance in the calibration group, compared to six excluded participants in the control group (Fisher’s exact test: *p* =.029). When inspecting group level performance in the objective measure, performance in the calibration group was not different from chance (M = 50.87%, SD = 2.06; *t*(19) = 1.89, *p* =.074, though BF₁₀ = 1.026), while performance was above chance in the control group, even after participant exclusion (M = 51.74%, SD = 2.02; *t*(19) = 3.86, *p* =.001; BF₁₀ = 35.04).

**Fig. 5:**
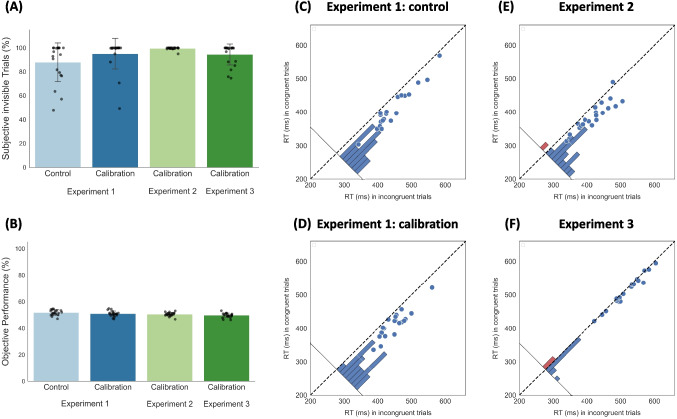
Results of Experiments 1, 2, and 3. *Top left* **(A)** Percentage of subjectively invisible trials in Experiment 1 (for the calibration and the control groups), Experiment 2, and Experiment 3. Each bar represents the group mean, with error bars reflecting the standard deviation (SD). Individual participant values are overlaid as *jittered black dots*. Bottom left **(B)** Performance in the objective test (2AFC) on subjective invisible trials in Experiment 1, 2, and 3, with the same conventions. *Right panels*: Reaction times (RTs) in congruent and incongruent trials for the **(C)** calibration group and **(D)** control group in Experiment 1, **(E)** Experiment 2, and **(F)** Experiment 3. Each *dot* represents the average RT of a single participant, with the *x*-axis corresponding to the RTs in incongruent trials and the *y*-axis to RTs in congruent trials. The *dashed diagonal line* marks equal RTs for both conditions, such that *dots below the line* indicate faster RTs for congruent trials, while those above indicate faster RTs for incongruent trials. The histogram at the bottom-left corner of each plot sums the number of dots with respect to the solid diagonal line

A priming effect was observed in both groups. In the calibration group, RTs were faster for congruent (M = 0.41 s, SD = 0.04) than for incongruent trials (M = 0.45 s, SD = 0.04; F(1, 19.19) = 71.17, *p*_corrected_ <.001; Fig. 5). An ITA was also found (Indirect d'—Direct d' = 0.15, SD = 0.22, *Ζ* = 3, *p* =.003, 95% CI [0.05, 0.25]), strengthening the claims for unconscious priming. The control group showed a similar pattern: congruent (M = 0.41 s, SD = 0.06) vs. incongruent (M = 0.44 s, SD = 0.06; *F*(1, 18.57) = 102.44, *p*_corrected_ <.001; Fig. 5). An ITA was again found (Indirect d'—Direct d' = 0.15, SD = 0.27, *Z* = 2.5, *p* =.012, 95% CI [0.04, 0.27]). To test whether the priming effect differed between the calibration and control groups, we fit an LMM and examined the interaction between Group and Condition. The model included fixed effects of Condition (congruent/incongruent; with random slopes and intercepts), Group (calibration/control), and their interaction. The main effect of Condition was observed (*F*(1, 38.06) = 166.71, *p* <.001), but we did not find a main effect of Group (*F*(1, 37.97) = 0.07, *p* =.81), nor an interaction (*F*(1, 38.06) = 0.0006, *p* =.98). Results were unchanged when including the six previously excluded participants (we again observed a main effect of Condition; *F*(1, 44.12) = 177.37, *p* <.001, with no main effect of Group; *F*(1, 44.01) = 0.12, *p* =.73, and no interaction between Group and Condition; *F*(1, 44.12) = 0.01, *p* =.90).

Additionally, since participants in the control group also performed the STEP procedure (although the ISI was fixed at 25 ms in the main task), we were able to examine their estimated thresholds. Across 26 participants, 13 participants had estimated thresholds below 25 ms, which was the threshold used for this group: of those, five were indeed excluded due to above-chance performance. In contrast, only one exclusion occurred among the 13 participants with thresholds ≥ 25 ms.

### Experiment 2

Experiment 2 replicated the results of Experiment 1, this time only focusing on the calibration group (mean estimated ISI = 26.67 ms, SD = 21.67). Here, trial exclusion was substantially minimized. In almost all trials (M = 99.59%, SD = 1.11), participants reported not seeing the prime, with objective performance not different from chance in these trials (M = 50.48%, SD = 1.42; *t*(19) = 1.52, *p* =.145, though BF₁₀ = 0.625). Note though, that here, two participants were excluded due to above-chance performance. Additionally, a congruency effect was found, with faster RT in congruent trials (M = 0.39 s, SD = 0.04) compared to incongruent trials (M = 0.42 s; SD = 0.04, *F*(1, 19.01) = 42.05, *p*_corrected_ <.001, BF₁₀ = 62.77; Fig. 5). Here too, an ITA was also found (Indirect d'—Direct d' = 0.11, SD = 0.18, *Z* = 2.75, *p* =.006, 95% CI [0.03, 0.19]). These results replicate the main findings of Experiment 1 and further support the reliability and effectiveness of the STEP calibration method for identifying upper subliminal thresholds suitable for unconscious processing research.

### Experiment 3

The mean estimated contrast was 0.42 (SD = 0.2). In most trials, participants were subjectively unaware of the prime (M = 94.5%, SD = 8.72). Additionally, participants’ performance in discriminating the prime did not differ from chance in these trials (M = 49.64%, SD = 1.69; *t*(19) = – 0.96, *p* =.34; though BF₁₀ = 0.349). A congruency effect was observed: participants responded faster on congruent trials (M = 0.51 s, SD = 0.04) than incongruent trials (M = 0.52 s, SD = 0.04; *F*(1, 9720.1) = 13.86, *p*_corrected_ <.001; Fig. 5). Akin to the previous analyses, an ITA was again found (Indirect d'—Direct d' = 0.1, SD = 0.18, *Z* = 2.75, *p* =.006, 95% CI [0.03, 0.18]). Thus, STEP calibration can also be used when contrast, rather than ISI, is manipulated, across different stimuli and masking protocols.

## Discussion

Here, we introduce STEP: a novel variant of the staircase calibration method, specifically tailored to target upper subliminal thresholds. Our simulations showed that when all metrics are considered, STEP outperforms not only the calibration procedures currently used in unconscious processing research, but also standard and modern psychophysical methods such as the Staircase (Levitt, 1971), PEST (Taylor & Creelman, 1967), QUEST (Watson & Pelli, 1983), QUEST + (Watson, 2017), and ASA (Faes et al., 2007) algorithms. Unlike these methods, which were developed and tested on estimating liminal or supraliminal thresholds, the STEP method particularly targets upper subliminal thresholds, where stimulus intensity is fine-tuned until participants reach chance-level performance, and is also nonparametric, thus requiring less assumptions. This provides a strong tool for studies of unconscious processing. In such studies, it is crucial to ensure that the stimuli of interest are presented subliminally while evoking the strongest signal possible, to avoid both over- and underestimation of unconscious effects. This requires an individual calibration procedure that is suited for identifying upper subliminal thresholds, so as to avoid residual awareness of the stimuli (which is commonly treated by trial and participant exclusion, known to have potential detrimental effects; Shanks, 2017), or overly suppressed stimuli that might preclude unconscious processing, even when it exists.

Furthermore, we conducted three experiments which further supported the effectiveness of STEP in empirical settings; Participants’ estimated thresholds were not found to be above chance-level performance in the objective tasks. Crucially, objective awareness was assessed using highly powered tests (480, 640, and 576 valid trials in Experiments 1–3, respectively), mitigating concerns about insufficient statistical power in detecting above chance performance (Vadillo et al., 2016, 2020; Yaron et al., 2024). Importantly, STEP resulted in extremely low trial and participant exclusion: in Experiment 1, no participant in the calibration group had to be excluded for above chance performance, compared to six excluded (23.08%) participants in the control group; in Experiment 2, only two participants (9.1%) were excluded, and one was excluded (4.76%) in Experiment 3. Given these encouraging indications, one might worry that the calibration was too conservative, pushing participants well below their thresholds. According to this account, no (or very weak) priming effects should be found. Notably, the opposite was true: despite participants’ convincing lack of awareness, we found unconscious priming effects in all three experiments, confirmed both when testing the effect directly and when using the ITA approach (Meyen et al., 2022).

These results demonstrate that the STEP procedure can be suitable for different experimental designs, using different types of stimuli, calibrating over different factors (here, both ISI and contrast), and using different masking protocols. STEP is also suited for experiments in which stimulus intensity is manipulated through other temporal parameters, such as stimulus duration. Unlike other methods (e.g., QUEST, QUEST +, PEST, ASA), which often require very small, continuous step sizes that are impractical when durations must be incremented in fixed frame units (constrained by the screen's refresh rate), STEP is designed to work within these discrete constraints. Note that we focused on masking because it is currently the most widely used method in the field (e.g., in the UnconTrust database, 72.77% of the experiments used masking; Schreiber et al., 2025). Future studies could test if STEP can be reliably applied to other suppression methods (e.g., Continuous Flash Suppression; Tsuchiya & Koch, 2005; or stimulus degradation; Kunst-Wilson & Zajonc, 1980), though thus far, calibration was less widely used with such methods (for exceptions see: Benthien & Hesselmann, 2021; Handschack et al., 2022, 2023). In addition, our proposed calibration method can also be used in studies that only rely on objective measures, in cases where subjective awareness measures are impractical or undesirable (see Schmidt, 2015; Stockart et al., 2025 for further discussion). Crucially, while STEP’s performance is somewhat reduced when it is used solely with objective measures (as shown in Supplementary Materials 5), it still outperforms the other tested methods across the different metrics we tested.

An additional advantage of STEP is that it does not require parametric assumptions. That is, the method does not rely on a predefined parametric template for the psychometric function (e.g., the cumulative normal, the Weibull, or the logistic functions), which has free parameters that are estimated (namely the threshold and the slope; Treutwein, 1995). Instead, STEP adapts purely based on participant performance, with the only assumption being that the curve is monotonically increasing. The field of unconscious processing typically does not use parametric calibration methods (Benthien & Hesselmann, 2021; Blakemore et al., 2017; Faivre et al., 2019; Handschack et al., 2023; Hung et al., 2016; Kiepe & Hesselmann, 2024; Lamy et al., 2008; Peel et al., 2018; Tal & Mudrik, 2024): If the assumptions about the psychophysical functions are violated (for instance, if the true psychometric function has an unexpected form), parametric methods might result in biased or unreliable estimates. This might explain why the QUEST method produced inaccurate estimates in our simulations. Using a nonparametric approach avoids the risk of making potentially unjustified assumptions. Furthermore, advanced parametric methods are often computationally expensive, more complex, and have fewer available implementations, which might render them less useful for researchers without specialized knowledge or software. In contrast, STEP was designed to be simple and easy to implement.

However, our method is not devoid of limitations. First, the benefits of using calibration generally come at a cost; Calibration procedures add more technical and procedural complexity by extending the duration of the experiment. This translates into spending more resources (namely, time and participant compensation) and may lead to fatigue and reduced attention in the main task (Boksem et al., 2005). Nonetheless, we argue that these short-term costs are outweighed by long-term gains: ultimately, STEP might require fewer resources since it reduces participant exclusion rate (hence less participants need to be run to obtain the desired sample size). Scientifically, the calibration procedure minimizes post hoc selection of both trials and participants, reducing bias (Fahrenfort et al., 2024; Shanks, 2017; Yaron et al., 2024). The reduced trial exclusion also increases power, as more unaware trials are included in the analysis.

Second, our solution is engineering-based, rather than mathematically based. That is, in theory, with an infinite number of trials, STEP will converge on a subliminal threshold, but this threshold might not be the exact ‘limen’ of subliminal threshold. This is because there is no principled mathematical criterion we are aware of that distinguishes the *upper subliminal threshold* from other subliminal thresholds that are lower than the limen. In both cases, performance will be at chance level. STEP addresses this inherent difficulty by starting the calibration at a supraliminal intensity and proceeding until strong evidence of chance-level performance is obtained (operationalized through our CWIR). This makes STEP practical and effective, despite not providing a purely mathematical solution to the problem of detecting the limen of conscious perception.

## Conclusion

In this paper, we sought to address a central methodological challenge in unconscious processing research: how to reliably calibrate stimulus intensity to ensure that it is presented subliminally while remaining strong enough to allow unconscious processing. We demonstrated that the calibration methods commonly used in the field are not effective in achieving this goal, either underestimating or overestimating the true upper subliminal threshold. Then, we introduced STEP, a novel nonparametric calibration method explicitly designed for unconscious processing studies. With a combination of both simulations and empirical validation, we showed that STEP outperforms the commonly used calibration methods in targeting the upper subliminal threshold, and that when using it, few participants are excluded while reliable effects are obtained. STEP’s generalizability and flexibility across masking techniques, stimulus manipulations, and awareness measures allow it to be widely adopted across different experimental paradigms. Incorporating this simple, reliable, and validated calibration procedure can enhance the reliability and robustness of findings in the field.

## Supplementary Information

Below is the link to the electronic supplementary material.

ESM 1 (DOCX 881 KB)

## Data Availability

All the collected data from all three experiments, and the preregistration for experiment 2, are available on OSF (https://osf.io/gq37p/files/osfstorage).

## Footnotes

1 Note that two additional participants were excluded: one who had above-chance performance (> 50%) across all levels and another who failed to reach higher performance (< 80%) at any level, implying he either did not follow instructions or did not fully perceive the stimulus even when it was presented at its highest strength.

## Code availability

All materials used for empirical analyses are available on OSF (https://osf.io/gq37p/files/osfstorage), and the simulation code is accessible via GitHub (https://github.com/EdenElbaz10/STEP-calibration).
