# Supplementary material for: The Subliminal Threshold Estimation Procedure (STEP): A calibration method tailored for estimating subliminal thresholds
Source: Behav Res Methods. 2025 Dec 1;58(1):13. doi: 10.3758/s13428-025-02872-3 (PMC12669343; doi:10.3758/s13428-025-02872-3)
Supplement: Supplementary file 1 — (DOCX 881 KB) [file 13428_2025_2872_MOESM1_ESM.docx]

**Supplementary Material**

1. **Prior sensitivity analyses:**

The priors for the Bayesian model for the objective ($\theta_{1}$, $\theta_{2};\mathrm{see} Equation 1)$ and subjective psychometric functions ($\theta, \sigma; \mathrm{see} Equation 2$) were modeled using a truncated normal distribution constrained to positive values. To ensure that the model accurately captured individual differences in the functions' parameters, we specified mildly informative normal priors for the ISI threshold of the objective psychometric functions ($\theta_{1}$) as $\mu=25$ and $\sigma=5$ (the value found to be most effective in suppressing the stimulus in Peremen & Lamy, 2014). The same priors as $\theta_{1}$were used for the remaining parameters, given that our results were not sensitive to prior specification:

**
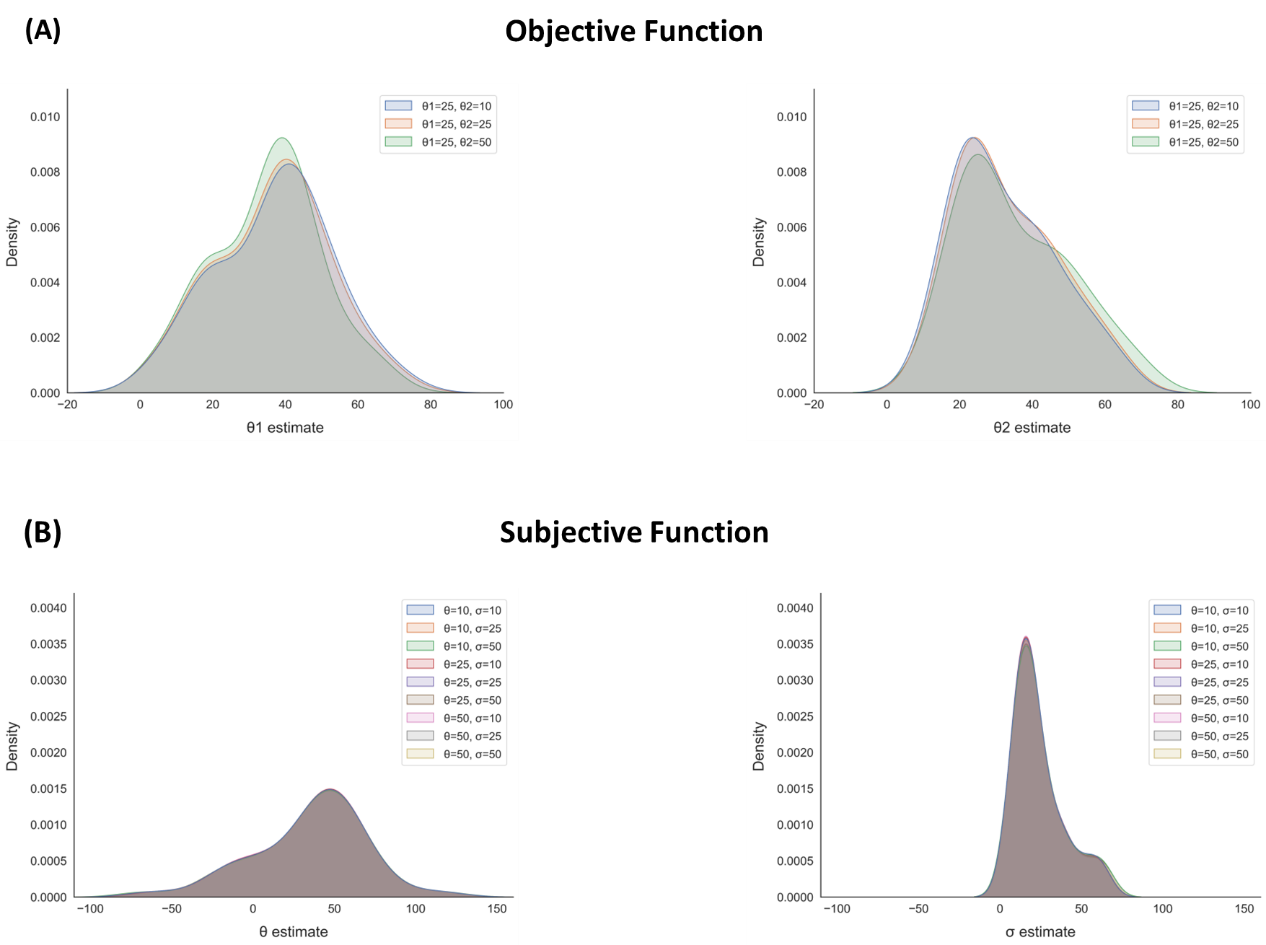
**

**Fig 1.** Distribution of individual estimates across different prior settings for (A) the objective psychometric function (2AFC): the estimated θ₁ (top left) and θ₂ (top right) values under three different prior settings (θ₂ = 10, 25, 50); and (B) the subjective psychometric function (PAS): the estimated θ (bottom left) and σ (bottom right) values under nine different prior settings combinations (θ = 10, 25, 50; σ = 10, 25, 50). Each line represents the kernel density estimate of the mean estimates across the 40 individuals who participated in the MOCS experiment, with different prior settings denoted by color. Despite variation in prior parameters, the resulting distributions largely overlapped, indicating that the final parameter estimates were not sensitive to prior specification.

1. **Testing calibration methods used in unconscious processing studies**

This section details the calibration procedures used in previously published unconscious processing studies that we examined (see Figure 1 in the main text). Where necessary, stimulus intensities were converted into comparable ISI-based durations.

| Paper | Method | Awareness measure | Step size | Stopping rule | Threshold calculation |
| --- | --- | --- | --- | --- | --- |
| Hung et al., 2023 | 1-up-1-down staircase | Objective | 3% of the current ISI | 40 trials | Final ISI |
| Peel et al., 2018 | 1-up-1-down staircase | Objective | 5% of the current ISI | 12 reversals | Mean of last 8 reversals |
| Biderman & Mudrik, 2018 | 1-up-1-down staircase | Objective | 15 ms* | 72 trials | second-lowest ISI |
| Tal & Mudrik, 2024 | 1-up-1-down staircase | Objective | 15 ms* | 100 trials | Second-lowest ISI  among last 40 trials |
| Bernstein et al., 1989 | 1-down staircase | Objective | 16.7 ms | 4 consecutive incorrect responses | Final ISI |
| Rothkirch & Hesselmann, 2018 | 1-up-1-down staircase | Subjective | Logarithmic (x0.7197) | 25 trials x 2 repetitions | Highest ISI consistently judged as invisible |
| Handschack et al., 2022 | 1-up-1-down staircase | Subjective | Logarithmic (x0.7197) | 20 trials | Highest ISI consistently judged as invisible |
| Kiepe & Hesselmann, 2024 | QUEST | Objective | - | 50 trials | Mode of posterior distribution |

We matched the procedures as closely as possible to the original specifications but had to introduce some modifications to allow the comparisons of methods; For Biderman & Mudrik (2018) and Tal & Mudrik (2024), the initial prime and mask contrasts were set to 0.7 and 0.85, respectively, and the step size was fixed at 0.05. In our simulations, we approximated this using a 15 ms ISI step size (~7% of the initial ISI), maintaining the same relative scaling.

For Kiepe & Hesselmann (2024), we implemented the QUEST algorithm (Watson & Pelli, 1983) using PsychoPy’s QuestHandler package. QUEST was initialized with an ISI of log10(100 ms) (corresponding to the average of the minimum and maximum ISI durations), and a prior SD of 1, allowing flexible sampling over plausible durations. The psychometric function was defined as a Weibull function with fixed parameters: slope β = 3.5 (default), lapse rate δ = 0.02, and guess rate γ = 0.5 (appropriate for a 2-AFC task). Since setting the target performance threshold (pThreshold) exactly to 0.5 is mathematically undefined within this formulation, the pThreshold was set to 0.51 to ensure convergence near chance level.

1. **Control analysis: testing calibration methods used in unconscious processing studies with 156 trials.**

As shown in the paper, the calibration methods previously used in unconscious processing studies were surprisingly inaccurate at estimating subliminal thresholds. One could claim, however, that this low performance might stem from the relatively small number of trials used (20-100). To test that, we ran an additional control analysis, increasing the number of trials to 156: For calibration methods with a fixed trial limit, we directly increased the number of trials to 156. For procedures with alternative stopping rule (e.g., Bernstein et al., 1989; Peel et al., 2018), we increased the number of repetitions to yield a comparable number of trials (four repetitions resulted in an average of 163.8 and 181.23 trials for the two papers, respectively). Despite this increase, performance remained suboptimal, underscoring the
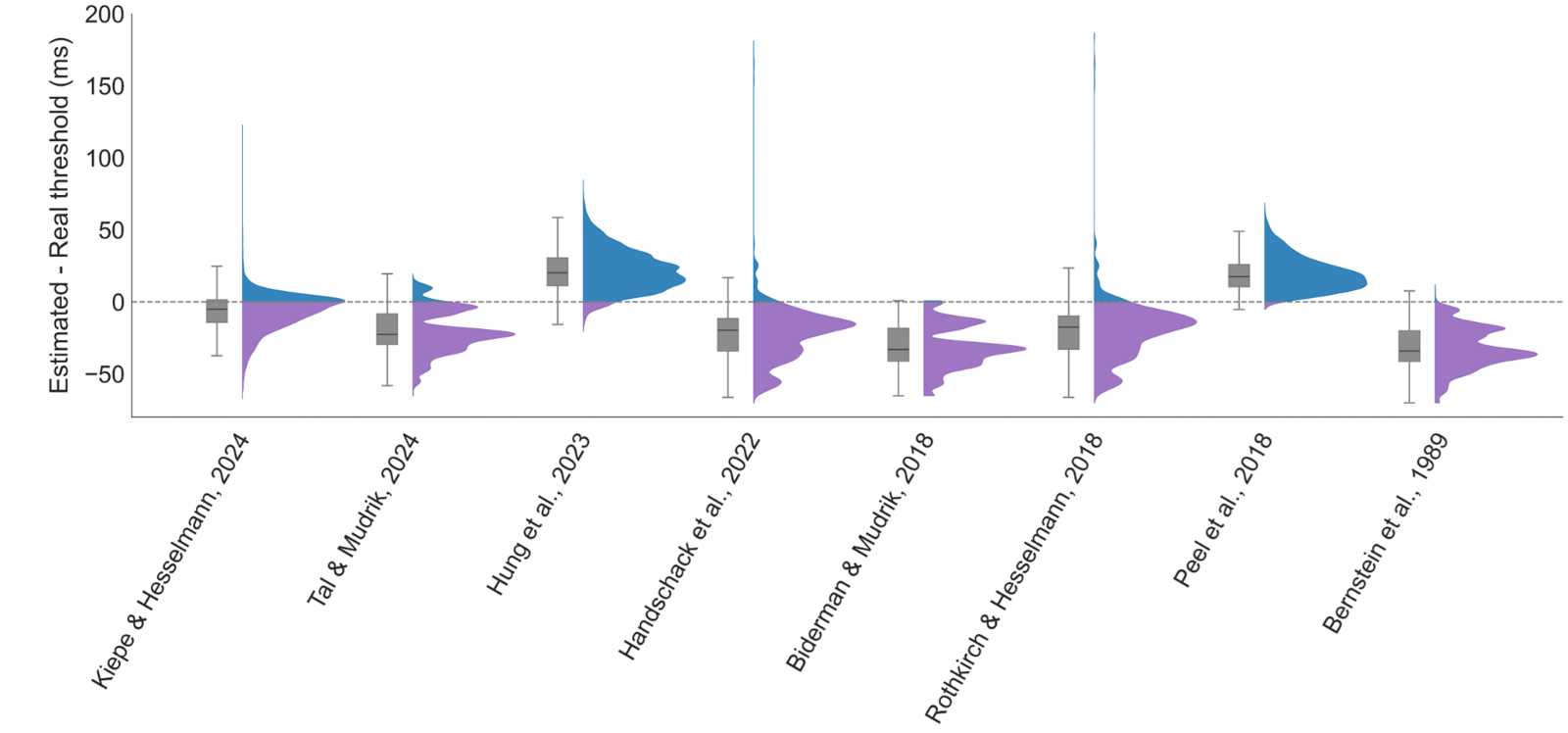
need for a calibration method specifically tailored to unconscious processing tasks (Figure 2).

**Fig 2.** Control analysis of calibration procedures used in eight unconscious processing studies with ~156 trials. Accuracy was assessed using simulations (10,000 virtual participants per method), and is presented as the threshold estimation error, the difference between the estimated and true subliminal threshold. Positive values indicate overestimation (in blue), negative values indicate underestimation (in purple). Accordingly, values closer to zero reflect greater accuracy, with the dashed line at zero indicating perfect threshold estimation. The x-axis lists study labels, and the y-axis indicates the density over threshold estimation error. Hence, the distribution for each study provides a full description of error values, including the central tendency and variability, thereby reflecting both the reliability (narrowness of distributions) and bias (systematic over- or underestimation) of each calibration approach. Overlaid gray boxplots represent the median, interquartile range, and overall spread.

1. **STEP objective variation:**

The STEP procedure can also be applied in studies that rely solely on objective measures, in cases where subjective awareness measures are impractical or undesirable. To adapt STEP for such use cases, two modifications are required: (A) Omit the **Integration with Subjective Measure** component. (B) For the **Upper Boundary**, instead of calculating it as a weighted average based on the seen ratio, define the new upper boundary as a simple average between the previous upper boundary and the current ISI at which significant performance was detected:

| $newBoundary =\frac{Boundary+currentISI}{2}$ | (1) |
| --- | --- |

**
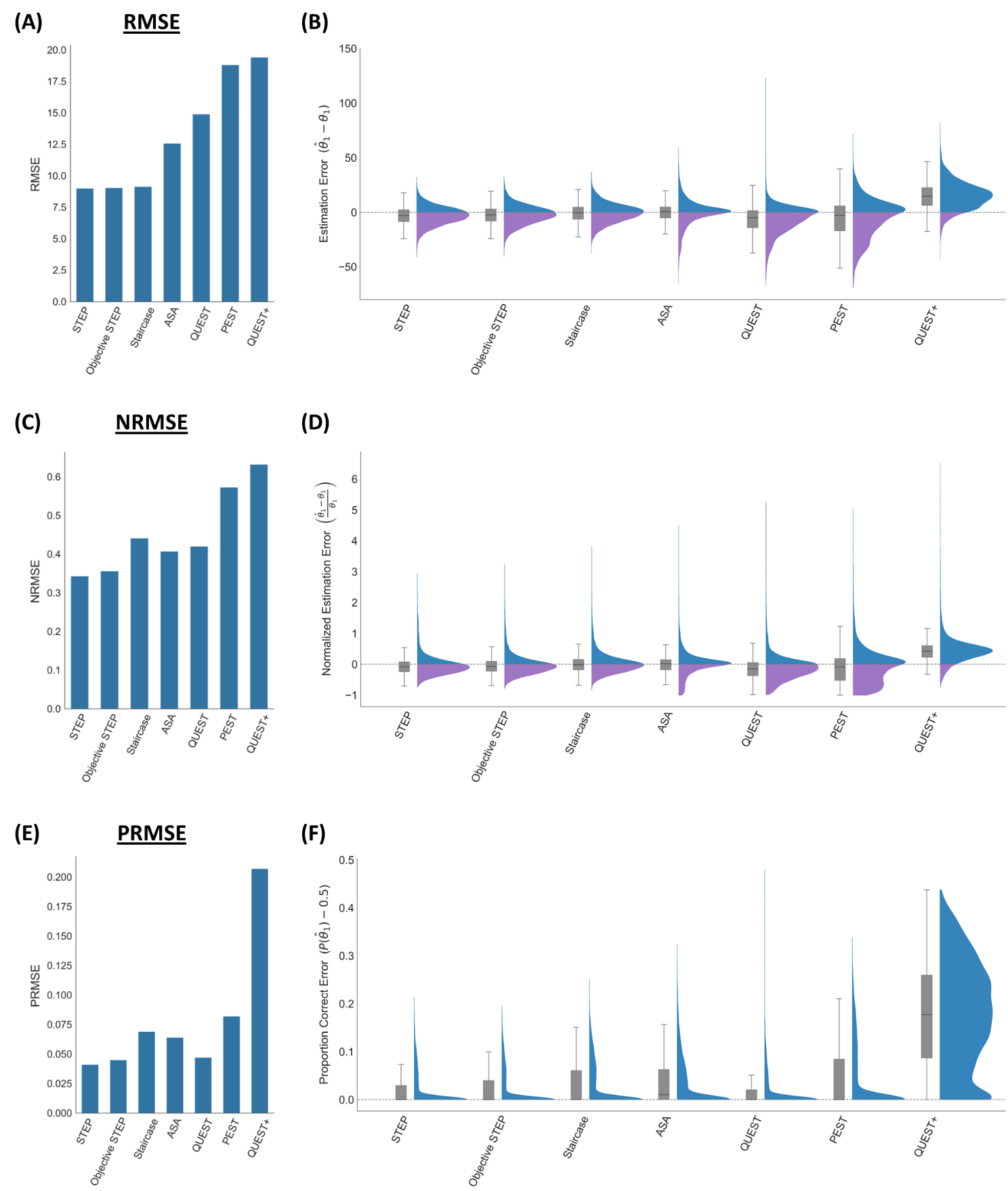
Simulation results including the objective variation of STEP:**

**Fig 3.** Accuracy of calibration procedures across three error metrics (lower values indicate better performance) and their corresponding distributions. **Top row:** Estimation error with (**A)** the RMSE values and **(B)** the difference between the estimated and true threshold ($\hat{\theta}_{1}$ - $\theta_{1}$). Positive values indicate overestimation (blue), negative values indicate underestimation (purple). Accordingly, values closer to zero reflect greater accuracy, with the dashed line at zero indicating perfect threshold estimation. The x-axis lists study labels, and the y-axis indicates the density over threshold estimation error. Hence, the distribution provides a full description of error values, including the central tendency and variability, thereby reflecting both the reliability (narrowness of distributions) and bias (systematic over- or underestimation) of each calibration approach. Overlaid gray boxplots represent the median, interquartile range, and overall spread; **Middle row:** relative error normalized by the true threshold with **(C)** NRMSE values and **(D)** Normalized estimation error (($\hat{\theta}_{1}$ – $\theta_{1}$)/ $\theta_{1}$). The same standards apply as in panel (B); **Bottom row:** Proportion correct error with **(E)** PRMSE (deviation of predicted performance from chance level) and **(F)** The deviance of the resulting performance of the estimated threshold in the objective measure and chance-level performance ($P$($\hat{\theta}_{1})$– 0.5). The same standards apply as in panel (B), though note that here, the difference cannot be negative since the minimal value of $P$($\hat{\theta}_{1})$ is 0.5. Across all metrics, although performance is somewhat reduced when using STEP with objective measures only, it still outperforms the other tested methods.

1. **Implementation and modifications for different calibration methods:**

To evaluate the accuracy of the STEP method, we compared its performance with several well-established calibration procedures commonly used in psychophysical research, including the staircase procedure (Levitt, 1971), Parameter Estimation by Sequential Testing (PEST; Taylor & Creelman, 1967), QUEST (Watson & Pelli, 1983), QUEST+ (Watson, 2017) (with some modifications), and the Accelerated Stochastic Approximation method (ASA; Faes et al., 2007; Kesten, 1958). To enable direct comparisons, the number of trials across all calibration methods was set to 156 to match the average number of trials used by the STEP procedure. Additionally, we used an adaptive adjustment, which allows a dynamic refinement of the step size estimates as the calibration progresses (Aleci, 2021; Leek, 2001). This is preferred over using fixed step sizes, which often limit exploration to a restricted range of the psychometric function, potentially biasing the threshold estimates (see again Aleci, 2021; Leek, 2001). Thus, the initial and maximal step size for ISI duration was 32 ms, with step-size reduction of 4 ms on each trial until reaching the minimal step size of 4 ms. Since the PEST, QUEST, QUEST+ and ASA methods require a higher resolution than 4 ms, there the step size was adaptively changed with no minimal step size.

The stopping rule was defined differently, according to the specific rules of each method. For QUEST, QUEST+, and ASA, sessions were terminated after a fixed number of trials, consistent with standard practice (Faes et al., 2007; Kingdom & Prins, 2016; Watson, 2017). Similarly, for PEST, the session typically ends either after a predefined number of trials or upon reaching the minimum step size (Aleci, 2021; Leek, 2001; Rinderknecht et al., 2018; Samaha et al., 2016). For all three methods, we set the stopping criterion to 156 trials. In contrast, the staircase procedure generally terminates after a specified number of reversals is reached (Garcı́a-Pérez, 1998; Kingdom & Prins, 2016). To ensure sufficient accuracy and reduce bias, it is recommended that at least 30 reversals be collected, with threshold estimates based on a stable portion of reversals (García-Pérez, 2000). We used a stopping rule of 35 reversals, and repeated the procedure twice, to allow the comparison with STEP, which includes two repetitions. The final threshold was then defined as the average of the estimates obtained in the two repetitions.

Similarly, the resulting estimated threshold was determined differently in each procedure; For ASA, this threshold was defined as the stimulus level that would have been presented after the last trial if the staircase had continued (Faes et al., 2007). For QUEST, the final threshold was estimated as the peak (mode) of the posterior distribution derived from participants' responses (Watson & Pelli, 1983): The procedure was identical to that used in the simulations for Kiepe & Hesselmann (2024) (for an additional implementation detail, see Supplementary Materials 2). For QUEST+, the final threshold was estimated using the mean of the posterior distribution across a multidimensional parameter space (threshold, slope, lapse rate, and lower asymptote). For PEST, the estimated threshold was the last stimulus level tested (Aleci, 2021). For the staircase method, the threshold was estimated as the mean of the stimulus levels at the reversal points, excluding the first two reversals for stability (García-Pérez, 2000; Garcı́a-Pérez, 1998; Meese, 1995).

In addition, QUEST+ was implemented using the linear stimulus scale and entropy minimization in the questplus package (<https://questplus.readthedocs.io/en/latest/index.html>), with modifications to incorporate our custom objective psychometric function (see Equation 1 in the main text): Initially, and consistent with the other methods, the stimulus domain ranged from 0 to 200 ms (with 100 steps); the threshold parameter ranged from 0 to 200 (in 100 steps); the slope from 1 to 200 (100 steps); with fixed lapse rate (0.02) and lower asymptote (0.5). However, this configuration yielded highly inaccurate results. Therefore, we restricted the stimulus and threshold domains to 0-150 ms (100 steps), and the slope to 1-150 (100 steps).

1. **Results are qualitatively unchanged when including all participants in the empirical experiments**

Below we report the results of the empirical experiments when including all excluded participants.

**Experiment 1.** In the analysis reported in the main text, no participant had to be excluded due to performance differing from chance in the calibration group, compared to six excluded participants in the control group. When including these participants in the control group, the results remained qualitatively unchanged: objective performance was still above chance (M=53.78%, SD=4.53; t(25)=4.25, *p*<.001; BF₁₀=112.87). In addition, a priming effect was observed, with faster RTs for congruent (M=0.42 s, SD=0.07) compared to incongruent trials (M=0.46 s, SD=0.06; *F*(1, 25.01)=110.99, *p*_corrected_<.001; Figure 4).

**Experiment 2.** Including the two previously excluded participants yielded the same results: Participants reported not seeing the prime in the vast majority of trials (M=96.65%, SD=10.05), with objective performance still not significantly different from chance in these trials (M=51.60%, SD=3.88; t(21)=1.93, *p*=.067, BF₁₀=1.07). A congruency effect was found, with faster RT in congruent trials (M=0.39 s, SD=0.04) compared to incongruent trials (M=0.42 s; SD=0.04, F(1, 20.96)=48.52, *p*_corrected_<.001, BF₁₀=235.51; Figure 4).

**Experiment 3.** When including the one previously excluded participant, the results remained qualitatively the same: in most trials, participants were subjectively unaware of the prime (M=92.66%, SD=11.94). Additionally, participants’ performance in discriminating the prime did not differ from chance in these trials (M=49.93%, SD=2.11; t(20)=-0.16, *p*=.88; BF₁₀=0.23). A congruency effect was observed: participants responded faster on congruent trials (M=0.51 s, SD=0.04) than incongruent trials (M=0.52 s, SD=0.04; F(1,10016)=14.43, *p*_corrected_<.001; Figure 4).


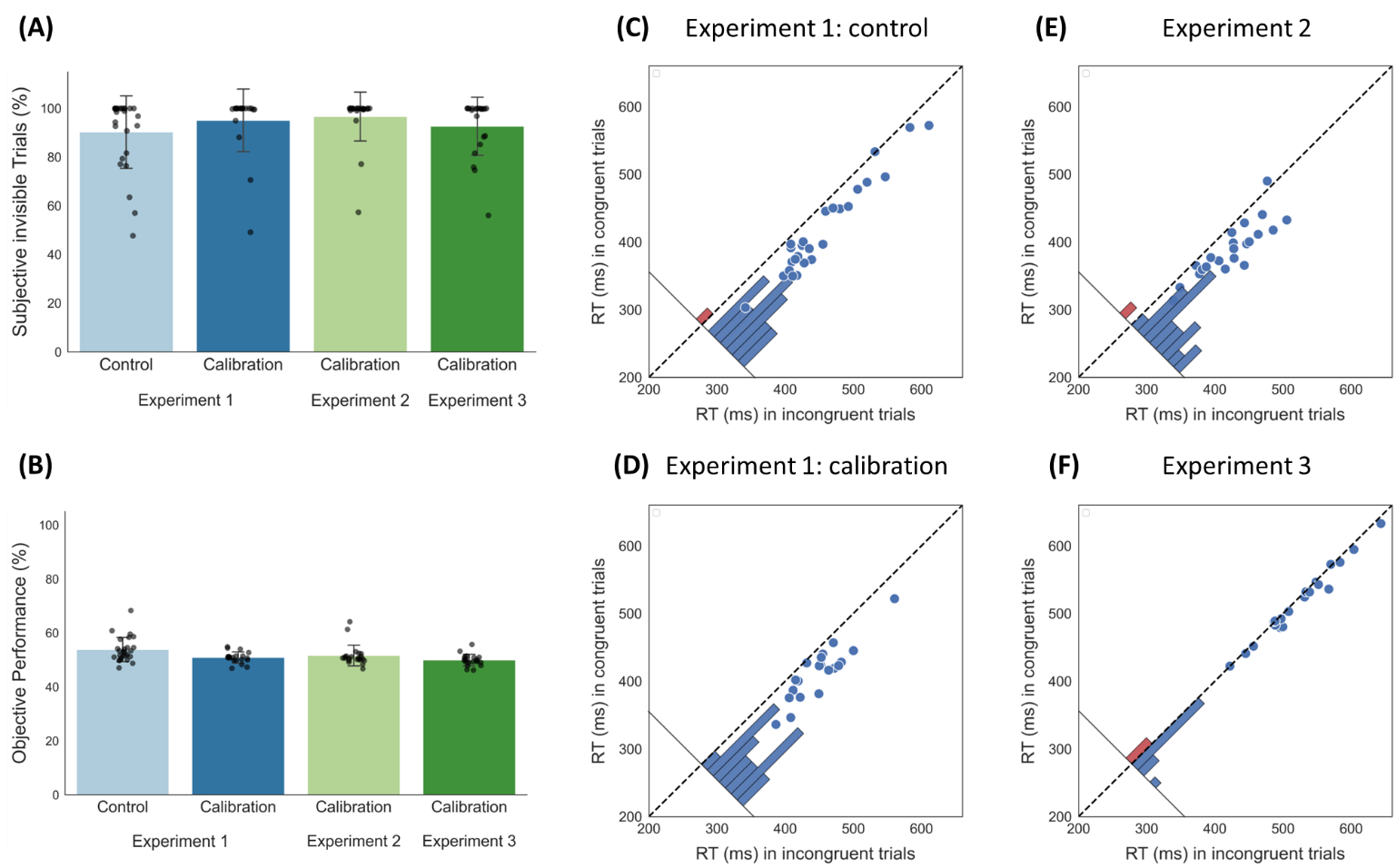


**Fig 4.** Results of Experiments 1, 2, and 3 when including the previously excluded participants. **Top left (A)**: percentage of subjectively invisible trials in Experiment 1 (for the calibration and the control groups), Experiment 2, and Experiment 3. Each bar represents the group mean, with error bars reflecting the standard deviation (SD). Individual participant values are overlaid as jittered black dots. **Bottom left (B)**: Performance in the objective test (2AFC) on subjective invisible trials in Experiment 1, 2, and 3 with the same conventions. **Right panels**: Reaction Times (RTs) in congruent and incongruent trials for the **(C)** calibration group (note that these results are identical to the main analysis, as no participants were excluded in this group) and **(D)** control group in Experiment 1, **(E)** Experiment 2 and **(F)** Experiment 3. Each dot represents the average RT of a single participant, with the x axis corresponding to the RTs in incongruent trials and the y axis to RTs in congruent trials. The dashed diagonal line marks equal RTs for both conditions such that dots below the line indicate faster RTs for congruent trials, while those above indicate faster RTs for incongruent trials. The histogram at the bottom-left corner of each plot sums the number of dots with respect to the solid diagonal line.

1. **Validation for low-level features in the face’s stimuli**

In a pilot experiment we conducted, an independent group of 16 participants (10 females and 6 males; age: M=28.69 years, SD=4.19) rated the valence of these images on a seven-point valence scale ranging from extremely negative to extremely positive. Based on their ratings, the 18 most extreme images for each emotional category (happy, fearful) and gender (male, female) were selected. The 72 selected images contained 54 unique identities, as nine female and nine male identities appeared once with a happy and once with a fearful expression (happy females: M=6.22, SD=0.25; fearful females: M=1.85, SD=0.26; happy males HA: M=6.02, SD=0.18; fearful males: M=1.78, SD=0.28).

The SHINE toolbox (Willenbockel et al., 2010) was used to assess and equate the luminance and contrast of all prime and target images; We applied SHINE’s imstats function to calculate the mean luminance and contrast of each of the 72 images. No difference in luminance or contrast was found between the fearful (luminance: M=91.32, SD=9.6; contrast: M=28.31, SD=3.24) and the happy expressions (luminance: M=91.08, SD=8.11; t(70)=0.11, *p*=.91, BF₁₀=0.24; contrast: M=28.65, SD=2.77; t(70)=-0.48, *p*=.63, BF₁₀=0.26).

**References**

Aleci, C. (2021). Chapter 9 Nonparametric Adaptive Psychophysical Procedures. In *Measuring the Soul: Psychophysics for Non-Psychophysicists* (pp. 45–68). EDP Sciences. https://doi.org/10.1051/978-2-7598-2517-2.c011

Bernstein, I. H., Bissonnette, V., Vyas, A., & Barclay, P. (1989). Semantic priming: Subliminal perception or context? *Perception & Psychophysics*, *45*(2), 153–161. https://doi.org/10.3758/BF03208050

Biderman, N., & Mudrik, L. (2018). Evidence for Implicit-But Not Unconscious-Processing of Object-Scene Relations. *Psychological Science*, *29*(2), 266–277. https://doi.org/10.1177/0956797617735745

Faes, L., Nollo, G., Ravelli, F., Ricci, L., Vescovi, M., Turatto, M., Pavani, F., & Antolini, R. (2007). Small-sample characterization of stochastic approximation staircases in forced-choice adaptive threshold estimation. *Perception & Psychophysics*, *69*(2), 254–262. https://doi.org/10.3758/BF03193747

García-Pérez, M. (2000). Optimal setups for forced-choice staircases with fixed step sizes. *Spatial Vision*, *13*(4), 431–448. https://doi.org/10.1163/156856800741306

Garcı́a-Pérez, M. A. (1998). Forced-choice staircases with fixed step sizes: Asymptotic and small-sample properties. *Vision Research*, *38*(12), 1861–1881. https://doi.org/10.1016/S0042-6989(97)00340-4

Handschack, J., Rothkirch, M., Sterzer, P., & Hesselmann, G. (2022). Probing the attentional modulation of unconscious processing under interocular suppression in a spatial cueing paradigm. *Cortex*, *153*, 32–43. https://doi.org/10.1016/j.cortex.2022.04.010

Hung, S.-M., Wu, D.-A., Escobar, L., Hsieh, P.-J., & Shimojo, S. (2023). Extracting probability in the absence of visual awareness. *Cognitive, Affective, & Behavioral Neuroscience*, *23*(3), 620–630. https://doi.org/10.3758/s13415-022-01057-1

Kesten, H. (1958). Accelerated Stochastic Approximation. *The Annals of Mathematical Statistics*, *29*(1), 41–59. https://doi.org/10.1214/aoms/1177706705

Kiepe, F., & Hesselmann, G. (2024). Prime-induced illusion of control: The influence of unconscious priming on self-initiated actions and the role of regression to the mean. *Consciousness and Cognition*, *121*, 103684. https://doi.org/10.1016/j.concog.2024.103684

Kingdom, F. A. A., & Prins, N. (2016). *Psychophysics: A Practical Introduction*. Elsevier Science & Technology. http://ebookcentral.proquest.com/lib/tau/detail.action?docID=4332363

Leek, M. R. (2001). Adaptive procedures in psychophysical research. *Perception & Psychophysics*, *63*(8), 1279. https://doi.org/10.3758/BF03194543

Meese, T. S. (1995). Using the standard staircase to measure the point of subjective equality: A guide based on computer simulations. *Perception & Psychophysics*, *57*(3), 267–281. https://doi.org/10.3758/BF03213053

Peel, H. J., Sperandio, I., Laycock, R., & Chouinard, P. A. (2018). Perceptual Discrimination of Basic Object Features Is Not Facilitated When Priming Stimuli Are Prevented From Reaching Awareness by Means of Visual Masking. *Frontiers in Integrative Neuroscience*, *12*, 13–13. https://doi.org/10.3389/fnint.2018.00013

Peremen, Z., & Lamy, D. (2014). Do conscious perception and unconscious processing rely on independent mechanisms? A meta-contrast study. *Consciousness and Cognition*, *24*, 22–32. https://doi.org/10.1016/j.concog.2013.12.006

Rinderknecht, M. D., Ranzani, R., Popp, W. L., Lambercy, O., & Gassert, R. (2018). *Method for Improving Psychophysical Threshold Estimates by Detecting Sustained Inattention* (p. 275594). bioRxiv. https://doi.org/10.1101/275594

Rothkirch, M., & Hesselmann, G. (2018). No evidence for dorsal-stream-based priming under continuous flash suppression. *Consciousness and Cognition*, *64*, 84–94. https://doi.org/10.1016/j.concog.2018.05.011

Samaha, J., Barrett, J. J., Sheldon, A. D., LaRocque, J. J., & Postle, B. R. (2016). Dissociating Perceptual Confidence from Discrimination Accuracy Reveals No Influence of Metacognitive Awareness on Working Memory. *Frontiers in Psychology*, *7*. https://doi.org/10.3389/fpsyg.2016.00851

Tal, A., & Mudrik, L. (2024). No evidence for unconscious initiation and following of arithmetic rules: A replication study. *Journal of Experimental Psychology. General*. https://doi.org/10.1037/xge0000622

Taylor, M. M., & Creelman, C. D. (1967). PEST: Efficient Estimates on Probability Functions. *The Journal of the Acoustical Society of America*, *41*(4A), 782–787. https://doi.org/10.1121/1.1910407

Watson, A. B. (2017). QUEST+: A general multidimensional Bayesian adaptive psychometric method. *Journal of Vision*, *17*(3), 10. https://doi.org/10.1167/17.3.10

Watson, A. B., & Pelli, D. G. (1983). QUEST: a Bayesian adaptive psychometric method. *Perception & Psychophysics*, *33*(2), 113–120. https://doi.org/10.3758/BF03202828

Willenbockel, V., Sadr, J., Fiset, D., Horne, G. O., Gosselin, F., & Tanaka, J. W. (2010). Controlling low-level image properties: The SHINE toolbox. *Behavior Research Methods*, *42*(3), 671–684. https://doi.org/10.3758/BRM.42.3.671/METRICS
